# Direct and indirect parental exposure to endocrine disruptors and elevated temperature influences gene expression across generations in a euryhaline model fish

**DOI:** 10.7717/peerj.6156

**Published:** 2019-01-08

**Authors:** Bethany M. DeCourten, Richard E. Connon, Susanne M. Brander

**Affiliations:** 1 Department of Environmental and Molecular Toxicology, Oregon State University, Corvallis, OR, United States of America; 2 Department of Biology and Marine Biology, University of North Carolina at Wilmington, Wilmington, NC, United States of America; 3 Department of Anatomy, Physiology and Cell Biology, University of California, Davis, CA, United States of America

**Keywords:** Ecotoxicology, Gene expression, Climate change, Endocrine disruption, Multiple stressors, Multigenerational, Temperature, *Menidia beryllina*, Bifenthrin, Ethinylestradiol

## Abstract

Aquatic organisms inhabiting polluted waterways face numerous adverse effects, including physiological disruption by endocrine disrupting compounds (EDCs). Little is known about how the temperatures associated with global climate change may influence the response of organisms exposed to EDCs, and the effects that these combined stressors may have on molecular endpoints such as gene expression. We exposed *Menidia beryllina* (inland silversides) to environmentally relevant concentrations (1 ng/L) of two estrogenic EDCs (bifenthrin and 17α-ethinylestradiol; EE2) at 22 °C and 28 °C. We conducted this experiment over multiple generations to better understand the potential effects to chronically exposed populations in the wild. We exposed adult parental fish (F0) for 14 days prior to spawning of the next generation. F1 larvae were then exposed from fertilization until 21 days post hatch (dph) before being transferred to clean water tanks. F1 larvae were reared to adulthood, then spawned in clean water to test for further effects of parental exposure on offspring (F2 generation). Gene expression was quantified by performing qPCR on F0 and F1 gonads, as well as F1 and F2 larvae. We did not detect any significant differences in the expression of genes measured in the parental or F1 adult gonads. We found that the 28 °C EE2 treatment significantly decreased the expression of nearly all genes measured in the F1 larvae. This pattern was transferred to the F2 generation for expression of the follicle-stimulating hormone receptor (FSHR) gene. Expression of 17β-hydroxysteroid dehydrogenase (17β-HSD) and G protein-coupled receptor 30 (GPR30) revealed changes not measured in the previous generation. Effects of the bifenthrin treatments were not observed until the F2 generation, which were exposed to the chemicals indirectly as germ cells. Our results indicate that effects of EDCs and their interactions with abiotic factors, may not be adequately represented by singular generation testing. These findings will contribute to the determination of the risk of EDC contamination to organisms inhabiting contaminated waterways under changing temperature regimes.

## Introduction

Many chemicals intended for human use, such as pharmaceuticals and pesticides, are found to pollute adjacent waterways and some are also known to disrupt endocrine function (Brander et al., 2013; Kuivila et al., 2012; Weston & Lydy, 2012; Brander et al., 2016a). Endocrine disrupting compounds (EDCs) influence hormone excretion and signaling in exposed organisms. For example, estrogenic EDCs can alter the expression of genes involved in reproduction and development (Scholz & Gutzeit, 2000; Tu et al., 2016; Brander et al., 2016b). These changes in gene expression may result in higher order consequences potentially impacting organismal function (Brander et al., 2013; Brander et al., 2016b). For example, the synthetic estrogen found in oral contraceptives, 17α-ethinylestradiol (EE2), has been shown to reduce egg production, influence sex ratios, and influence development in fishes exposed to environmentally relevant concentrations (Kidd et al., 2007; Vajda et al., 2008; Schwindt et al., 2014; Bhandari, Vom Saal & Tillitt, 2015). Many pesticides, such as the commonly used pyrethroid pesticide, bifenthrin, can produce estrogenic effects at low doses that deviates from their intended mode of action on the sodium channels of presynaptic neurons of target pests (Brander et al., 2013). Like EE2, bifenthrin has been shown to impact fishes at multiple levels of biological organization; e.g., gene expression, leading to impaired development and reproduction (Brander et al., 2016a; Brander et al., 2016b; Tu et al., 2016).

Estuaries are typically highly impacted by pollution because they are heavily used by growing coastal communities (Cloern & Jassby, 2012). Protection of estuaries is vital for maintaining healthy food webs and ecosystem services. The model species, *Menidia beryllina* (common name: inland silverside), is a small forage fish that is a food source to a number of estuarine taxa, including commercially important species. *Menidia* are tolerant of a wide range of salinities but are sensitive to chemical stressors, making them an excellent model species to test the effects of anthropogenic stressors that are common in estuaries (Hemmer, Middaugh & Moore, 1990; Brander et al., 2013). Past studies have shown that the expression of genes involved in reproduction, growth and immune function can be altered following EDC exposure in *M. beryllina*. *Menidia* species may be especially susceptible to increasing temperatures and pollutants as their sex determination can be influenced by both temperature and EDC exposure (Duffy, McElroy & Conover, 2009; DeCourten & Brander, 2017). Understanding how organisms with sensitive physiology, such as *M. beryllina*, respond to EDC exposure will help determine the potential risk of these stressors on estuarine ecosystems.

As our planet continues to warm at an unprecedented rate (Stocker et al., 2013), it becomes increasingly important to understand the impacts pollution may have in the context of global climate change. Increases in temperature can inhibit growth, reproduction and early-life stage survival of marine fishes (Munday et al., 2008; Pankhurst & Munday, 2011). Expression of genes along the hypothalamus-pituitary-gonad (HPG) axis can be reduced by exposure to increased temperatures, inhibiting gonadal function (Miranda et al., 2013). The impacts of increased temperature on fishes could lead to changes to overall community structure and trophic dynamics (Jeppesen et al., 2010). Studies suggest that the effects of estrogenic endocrine disruptors may be influenced by other abiotic factors such as ambient temperature (Hemmer, Middaugh & Moore, 1990; Luzio et al., 2016; DeCourten & Brander, 2017). Testing effects of EDCs under different temperature regimes will help determine the risk they pose to wildlife in the context of global climate change. Elevated temperature may exacerbate the effects of EDCs by increasing the uptake and metabolism of these chemicals in ectothermic organisms (Clarke & Fraser, 2004). Additionally, some chemicals such as bifenthrin, which is converted into a metabolite (4-hydroxybifenthrin), have more potent metabolites than their parent compounds (DeGroot & Brander, 2014). In this study, we investigated the effects of two estrogenic EDCs (EE2 and bifenthrin) on genes involved in development and reproduction of *M. beryllina*, at two temperatures: 22 °C and 28 °C, to understand how rising temperatures may influence the toxicity of these chemicals. The temperatures chosen for this study represent the most extreme possibility from the worst-case scenario temperature increase (RCP 8.5) expected by the year 2100, put forth by the International Panel for Climate Change (IPCC; Stocker et al., 2013). The RCP 8.5 model predicts a surface temperature increase of 1.4–2.6 °C between 2046 and 2065 and a 2.6–4.8 °C 2081–2100 (Stocker et al., 2013). Assuming these warming periods are additive, we might expect a 4–7.4 °C increase over the next century, illustrating the importance for testing a 6 °C temperature increase. This is especially important for organisms with plasticity in development, such as *M. beryllina*, whose sex determination can be influenced by temperature and EDC exposure independently (Duffy, McElroy & Conover, 2009; DeCourten & Brander, 2017). The 28 °C upper temperature limit was chosen to allow us to test for effects on TSD without reaching the upper breeding limit of *M. beryllina* (30 °C), at which spawning is curtailed (Hubbs, 1982). This experiment was conducted over three generations to determine effects at different life stages and effects of parental exposure on offspring. Understanding the effects of concurrent stressors across multiple generations on the gene expression of exposed organisms is necessary to determine the potential for long-term population impacts.

## Methods

### *Menidia beryllina* rearing and exposures

A subset of individuals used in a previous study (DeCourten & Brander, 2017) investigating the effects of elevated temperature and EDC exposure on reproduction, development and survival, were sampled for analysis of molecular endpoints. The following methods pertaining to fish rearing, exposures and water chemistry were initially described in DeCourten & Brander (2017). The parental generation of *M. beryllina* were obtained from Aquatic Biosystems (Fort Collins, CO, USA) as juveniles and reared to adulthood at 25 °C, at a salinity of 15 ppt at the University of North Carolina, Wilmington’s Center for Marine Science. Fish were fed Hikari tropical micro pellets (Kyorin Food Industries, Ltd, Japan) and live *Artemia nauplii* supplemented with Selcon™ (vitamin supplement; American Marine Inc., Ridgefield, CT), during rearing and experimental exposures. Adult fish (341 dph) were moved to experimental tanks and acclimated to either 22 °C or 28 °C for 3 weeks prior to experimental exposures under a continuous 16:8 light-dark cycle.

Parental exposures were initiated by replacing approximately 90 percent of the water in experimental tanks with water containing either 1 ng/L of bifenthrin (purity: 99.5%, Chem Service, West Chester, PA, USA) or 1ng/L 17α-ethinylestradiol (EE2) (purity: ≥98%, Sigma-Aldrich, St. Louis, MO, USA) suspended in 1 µL/L of methanol due to the hydrophobic nature of the EDCs. We used 1 µL/L concentration of methanol in our control group to account for any adverse effects that may be attributed to the solvent. Adult fish (341 dph) were exposed for 14 days in 7.6 L glass jars, containing approximately 6 L of artificial seawater. Exposure jars were housed in 60 L water baths fitted with aquarium heaters set to maintain experimental temperatures. Fish were maintained at a constant salinity target of 15 ppt; optimal for growth and survival of *M. beryllina* (Middaugh, Hemmer & Lamadrid-Rose, 1986). Artificial seawater was made daily by adding Instant Ocean (Spectrum Brands, Blacksburg, VA) to distilled water, which was aerated for 24 h prior to use. Solutions with experimental EDCs were mixed in glass containers immediately prior to daily water changes. Jars were continuously aerated and water quality parameters including temperature (21.9 °C ± S.D. 0.41 and 27.7 °C ± S.D. 0.89, respectively), dissolved oxygen (5.81 ± S.D. 0.98), pH (7.94 ± S.D. 0.56), salinity (16.26 ± S.D. 1.59), and ammonia (0.22 ± S.D. 0.76) were measured with a YSI Professional Plus Quatro water quality meter daily before and after water changes in two representative control jars at each temperature. Water bath temperatures were measured once daily throughout the duration of the experiment. Debris was removed, and sixty percent of the water replaced daily in experimental chambers. All air pumps and heaters were connected to an emergency power source to ensure continuous function. Adult fish were fed a diet of Hikari fish food pellets, freeze-dried blood worms and live *Artemia nauplii* supplemented with Selcon™. Larvae were fed live rotifers (*Brachionus rotundiformis*) from 0–14 dph, and a combination of live *Artemia* and ground Hikari pellets from 10 dph to adulthood. Fish were fed daily and allowed to feed for one hour prior to water change. Following exposures, spawning trials were initiated with the parental generation adults.

Spawning trials were initiated by the addition of spawning substrate (6-inch strands of bleached and rinsed dye-free yarn), as described in Brander et al. (2016b). The fish were allowed to spawn for 48 h to produce the subsequent generation. Following the spawning period adult fish were euthanized in an overdose solution of buffered tricaine methanesulfonate (MS-222; approximately 200 mg/L) and immediately weighed and measured, and gonads of three males and three females were collected from each replicate jar. Spawning substrate with attached embryos remained in experimental jars allowing embryos to hatch without transfer. F1 larvae were reared with chemical exposure until 21 dph at which point a subset of five larvae were sampled from each of the 5 replicate jars, to be used for gene expression analysis. Sampled adult tissue and 21dph larvae were immediately frozen in liquid nitrogen and stored at −80 °C. All remaining fish were transferred to clean 37.5 L round black polypropylene tanks and reared to maturity at either 22 °C or 28 °C. Each tank was equipped with Marineland Penguin PF0100B filter, one Eheim Jager aquarium thermostat heater (75 watt) and Resun AF-2009D automatic feeder. F1 adults were spawned, and the F2 larvae produced were then reared and spawned in clean water (without EDC contamination) at experimental temperatures until 21 dph, to test the effect of parental exposure on offspring. At this time, all larvae were sampled, and the experiment was terminated. All experiments were conducted in accordance with UNCW Institutional Animal Care and Use protocol #A1314-010 and #A1415-010.

### Analytical chemistry

Water samples for analytical chemistry were made using the same methods used to make water for the experimental treatments, poured into 950 mL sterile amber bottles, transported on wet ice and stored in the dark at 4 °C for no longer than 24 h. Extraction was performed using conditioned 6 mL solid phase-extraction C_18_ cartridges (Superclean™ 500 mg; Sigma Aldrich) at a slow drip. Columns were rinsed twice with 5 mL of a 1:1 solution of hexane:ethyl acetate to elute the bifenthrin. The solvent elution (10 mL) was concentrated to 0.4 mL under a gentle stream of nitrogen. Final extract was analyzed using gas chromatography negative chemical ionization mass spectrometry (GC-NCI-MS) on an Agilent 5973 series gas chromatograph (Agilent Technologies, Palo Alto, CA, USA) equipped with a split-splitless injector (280 °C, splitless, 1.5 min purge time). The columns used were Supelco DB-5MS column (30 mm × 0.25 mm with a 0.3 µm film thickness). The instrument was calibrated using nine sets of standard bifenthrin solutions, the surrogate trans-permethrin D6 (EQ Laboratories, Atlanta, GA, USA), and the internal standard dibromooctafluorobiphenyl (Chem Service, West Chester, PA, USA) in hexane. Controls were conducted by analyzing a method blank of deionized water (Milli-Q) to ensure samples were not contaminated. A surrogate was added to each sample before extraction to monitor matrix effects and method performance. The detection limit of bifenthrin in whole water was 0.6 ng/L. No bifenthrin was detected in the controls or method blanks. Measured concentrations of bifenthrin were 1.3 ng/L. Surrogate recovery was 133.84%, with corrected value of bifenthrin concentration being 0.89 ng/L. EE2 treatment water was prepared, in a similar matter to the bifenthrin, according to previously established protocols (DeGroot & Brander, 2014; Brander et al., 2016b). All stock and experimental solutions were mixed in the same manner as bifenthrin to ensure like concentrations.

### Gene expression quantification

A random subset of 2–3 larvae and 1–2 male and female gonads were selected from each experimental replicate (*n* = 3–5) and used for quantification of gene expression (Table S1). RNA was extracted using RNeasy Mini Kits (Qiagen, Hilden, Germany) Total RNA concentration and 260/280 ratios (acceptable values > 1.6) were quantified using a Nanodrop 1000 spectrophotometer (ThermoFisher Scientific, Waltham, MA). RNA purity was assessed via electrophoresis on a 1% agarose gel and visualized on a Gel Doc™ XR + Gel Documentation system (BioRad, Hercules, CA, USA). Samples containing trace amounts of genomic DNA were treated with DNase I (Invitrogen™, Waltham, MA) and cleaned up using RNeasy MinElute Cleanup Kit (Qiagen, Hilden, Germany). RNA concentrations were adjusted to a concentration of 100 ng/µl in 96-well plates, randomizing samples within each generation and tissue type prior to cDNA synthesis. Complimentary DNA (cDNA) was synthesized using Superscript III (Invitrogen, Carlsbad, CA, USA), following manufacture protocols and methods in Jeffries et al. (2015). Twelve genes were selected for quantitative polymerase chain reaction (qPCR), including two reference genes (Table 1). We chose to examine a suite of genes involved in reproduction, growth and development based on previous findings from DeCourten & Brander (2017), as well as Brander et al. (2016b). Within the larval samples we chose genes primarily involved in steroidogenesis (17β-HSD, 3β-HSD, CYP19b), reproductive pathways (ERs, ARx, FSHR, GnRHR) and growth (IGF2) due to the differences in sex ratio and developmental deformities after early life stage exposure observed in our previous study (DeCourten & Brander, 2017). Within the adult gonadal tissues, we selected genes involved in reproductive pathways (ERs, ARx, FSHR, HSP90), and steroidogenesis (17β-HSD, 3β-HSD, CYP19a, INHA) also due to our previous observations of skewed sex ratios and reduced reproductive success (DeCourten & Brander, 2017). Larval cDNA samples were diluted 2:3 and adult gonad samples were diluted 1:4 with nuclease free water to optimize the reaction. We conducted qPCRs using previously established protocols in Brander et al. (2016b), with Maxima Probe/ROX qPCR Master Mix (ThermoFisher Scientific). Primers and probes were obtained from Eurofins Genomics (Louisville, KY) and Roche LifeScience (Indianapolis, IN), respectively. We conducted qPCRs using 8 µL cDNA and 12 µL of mastermix containing 83.3% Maxima Probe/ROX qPCR Master Mix (Thermo Fisher Scientific), 8.3% primers, and 8.3% probe. qPCRs were run on a BioRad CFX96. Reaction protocols included a 10 min hot start incubation at 95 °C, and 40 cycles of 15s at 95 °C, and 30s at 60 °C, during which optical data for the plate was recorded. The environmentally relevant concentrations used elicit small changes in gene expression, thus high concentrations of cDNA were required. Technical replication (x3) was conducted for qPCR efficiency tests when validating primers which demonstrated a high level of accuracy between replicates. Efficiency values for the majority of genes used in this study ranged from 95.1%–107.8%, with the exception of CYP19b (119.2%) and INHA (123.6%), both of which have been used successfully in a previous study (Brander et al., 2016b). We did not run technical replicates for experimental qPCRs due to the small amount RNA available from small tissue and larval samples. We opted instead to have a greater number of gene responses examined instead of including technical replicates as previously done in Connon et al. (2011), Connon et al. (2012), Jeffries et al. (2015), Brander et al. (2016b), Frank et al. (2019) and Goff et al. (2017). Protocols were developed based on manufacturer’s recommendations and previously established protocols from Brander et al. (2016b).

**Table 1:** Genes used for qPCR analysis. Information on genes selected for qPCR assays.

Gene	Symbol	Roche UPL #	Primer	Function
17-beta-hydroxysteroid dehydrogenase 14	HSD17B14	142	F: gatctgctcaacctcaatcttgtc	Steroid synthesis
			R: caggtgtggcaacgcaaat	
3-beta-hydroxysteroid dehydrogenase delta 5	HSD3B2	133	F: caccatgctcaacaccacctt	Glucocorticoid and androgen synthesis
			R: ctcgtaccccaggtctttcttg	
Gonadal aromatase *(adults only)*	CYP19a	3	F: gcctcccacagaccaacaat	Steroid metabolism
			R: gccatgctgaggtgttcagtc	
Brain aromatase *(larvae only)*	CYP19b	25	F: cagaacccagatgtggagcag	Steroid metabolism
			R: cacagactttcccctgaacacc	
Estrogen receptor alpha	ESR1	15	F: ctccattgtgccagtgcaga	Hormone receptor
			R: acgcttccgcatgctca	
Estrogen receptor beta a	ESR2	52	F: gaccatcctgggaaactgatctt	Hormone receptor
			R: cattatgccctccacgcact	
Estrogen receptor beta b	ESR3	130	F: gattttattcaaccggagcagtg	Hormone receptor
			R: catcggctcgtctgatgaact	
Androgen receptor	Arx	31	F: atccgcatgcagtgctcata	Hormone receptor
			R: ccccagacctcgtattcaacg	
Follicle stimulating hormone receptor	FSHR	67	F: tgggatctcaacaatgccttc	Hormone receptor
			R: gatccgcgtctgcttaatttg	
Gonadotropin-releasing hormone receptor 1	GnRHR	108	F: cggtggcgtggacaatg	Hormone receptor
			R: ggtgatggtcacgttgtgga	
G-protien coupled estrogen receptor *(larvae only)*	GPR30	48	F: tcttcgtgctgctgttgtcat	Hormone receptor
			R: cgtcctctccggcctctac	
Insulin-like growth factor 2 *(larvae only)*	IGF2	38	F: gcaggtcatacccgtgatgc	Growth factor
			R: ggctgccttcctattccacac	
Heat shock protein 90 beta *(adults only)*	HSP90AB1	55	F: gaaaaggtgaccgtgtccaac	Molecular Chaperone
			R: gccgtaagtgctggtcacaat	
Inhibin alpha subunit *(adults only)*	INHA	24	F: gaggaggatcagggggactg	Negative Feedback
			R: cgggtggacgatccacttt	
60s ribosomal protein l7	RPL_7	31	F: aacttcttgtggccgttcaag	Reference gene
			R: tcgcctccctccacaaagt	
Elongation factor 1-alpha-like	EF1a	65	F: catcgcctgcaagttcagc	Reference gene
			R: cccagacttcagcgcctt	

### Data analysis

Data was collected from 1–2 individual male and female gonads and 2–3 individual larvae in each replicate tank (*n* = 3–5). Thresholds were set using CFX manager software (BioRad) and calculated CT values were exported to Microsoft Excel 2016. CT values were normalized by the geometric mean of the two housekeeping genes (EF1a and RPL7) used in this study. Housekeeping genes were confirmed to be stable across treatments using GeNorm (Vandesompele et al., 2002). Normalized CT values were then converted to log2 fold change values using methods described in Livak & Schmittgen (2001). Further information is provided in Data S1. Values from multiple fish in each replicate were averaged to obtain one value for each replicate tank. We tested for significant differences (*p* < 0.05) among all treatments using a two-way ANOVA and Tukey’s post hoc multiple comparisons with false discovery rate *p*-value corrections applied (*p* < 0.05; Benjamini & Hochberg, 1995). All statistical tests were performed in R (R Core Team, 2017; v 3.4.3) and Tukey’s post hoc multiple comparisons were made using the *multcomp* package (Hothorn, Bretz & Westfall, 2008).

## Results

### Parental generation

There were no significant differences in expression of genes tested in the parental generation adult ovaries (Fig. S1) or testes (Fig. S2) following experimental exposures. A full list of pairwise comparisons and *p*-values across all generations is presented in Table S2.

**Figure 1:**
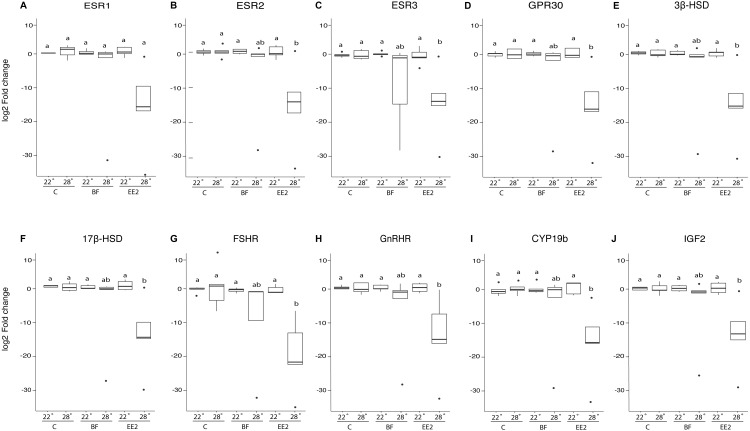
Relative gene expression of F1 larvae. Gene expression in fold change (log2) of the ESR1 (A), ESR2 (B), ESR3 (C) GPR30 (D), 3β-HSD (E), 17β-HSD (F), FSHR (G), GnRHR(H), CYP19b (I), and IGF2 (J) genes. Values normalized to the geometric mean of the housekeeping genes in the F1 larvae (21 dph) at 22 °C and 28 °C in the control (C), bifenthrin (BF) and ethinylestradiol (EE2) treatments. Boxes denote inner quartile range, whiskers represent upper and lower quartiles, and median is denoted by solid bars. Significance is denoted by lowercase letters (two-way ANOVA, Tukey’s HSD, *p* < 0.05, *n* = 5).

### F1 generation

Within the F1 larvae, (21 dph; Fig. 1) we found that the 28 °C EE2 treatment significantly reduced the expression of all genes except for ESR1, relative to all other experimental treatments with the exception of 28 °C bifenthrin. There was reduced expression of most of the estrogen receptors tested including: ESR2 (two-way ANOVA, Tukey’s HSD, *p* ≤ 0.05), ESR3 (two-way ANOVA, Tukey’s HSD, *p* ≤ 0.05) and GPR30 (two-way ANOVA, Tukey’s HSD, *p* ≤ 0.05), in the 28 °C EE2 treatment. There was reduced expression of receptors for hormones secreted by the pituitary: GnRHR (two-way ANOVA, Tukey’s HSD, *p* ≤ 0.05) and FSHR (two-way ANOVA, Tukey’s HSD, *p* ≤ 0.05). Expression of genes involved in steroidogenesis was reduced in the 28 °C EE2 treatment including: CYP19b, (two-way ANOVA, Tukey’s HSD, *p* ≤ 0.05), 17β-HSD (two-way ANOVA, Tukey’s HSD, *p* ≤ 0.05) and 3β-HSD (two-way ANOVA, Tukey’s HSD, *p* ≤ 0.05). We also determined a decrease in expression of IGF2 (two-way ANOVA, Tukey’s HSD, *p* ≤ 0.05), which is involved in growth regulation. For a full list of *p*-values for all pairwise comparisons refer to Table S2. There were no significant differences in gene expression of the F1 larvae among any of the 22 °C treatments, and we found there were no significant changes in gene expression in any of the genes tested in the adult F1 ovaries (Fig. S3) or testes (Fig. S4) in any of the chemical or temperature treatments.

### F2 generation

**Figure 2:**
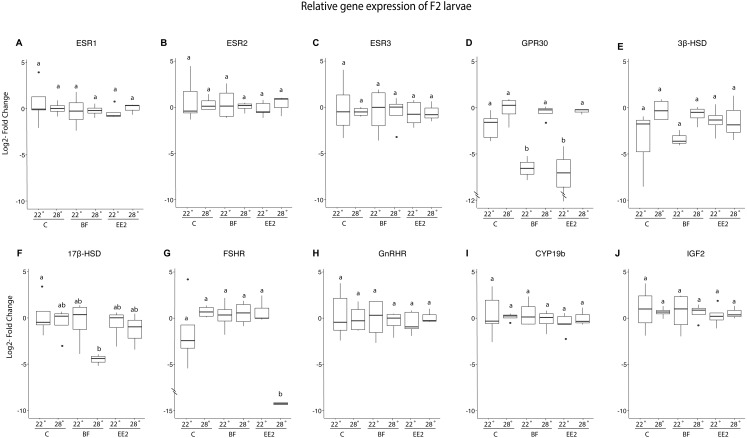
Relative gene expression of F2 larvae. Gene expression in fold change (log2) of the ESR1 (A), ESR2 (B), ESR3 (C) GPR30 (D), 3β-HSD (E), 17β-HSD (F), FSHR (G), GnRHR(H), CYP19b (I), and IGF2 (J) genes. Values normalized to the geometric mean of the housekeeping genes in the F2 larvae (21 dph) at 22 °C and 28 °C in the control (C), bifenthrin (BF) and ethinylestradiol (EE2) treatments. Boxes denote inner quartile range, whiskers represent upper and lower quartiles, and median is denoted by solid bars. Significance is denoted by lowercase letters (two-way ANOVA, Tukey’s HSD, *p* < 0.05, *n* = 5 in the 22 °C EE2 and 22 °C Control, *n* = 4 in 22 °C BF, 28 °C BF and 28 °C Control, *n* = 3 in 28 °C EE2).

Within the F2 larvae (Fig. 2), we found that GPR30 expression was reduced by the 22 °C bifenthrin and EE2 treatments (two-way ANOVA, Tukey’s HSD, *p* ≤ 0.01). There were no differences in expression in the other estrogen receptors (ESR1, ESR2, ESR3). The pattern of expression of FSHR observed in the F1 generation was carried over into the F2 generation (Fig. 2), with the 28 °C EE2 treatment significantly reducing the expression of this gene when compared to all other treatments (two-way ANOVA, Tukey’s HSD, *p* ≤ 0.01). We did not observe any differences in expression of GnRHR, indicating that receptors of hormones secreted by the pituitary responded differently in the F2 generation. The expression of 17β-HSD was significantly altered by the 28 °C bifenthrin treatment when compared to the 22 °C control (two-way ANOVA, Tukey’s HSD, *p* = 0.0249). Other genes involved in steroidogenesis (3β-HSD and CYP19b) in the F2 generation were not significantly affected by the experimental exposures. As observed in the previous generation, we did not observe differences in expression of IGF2 among the experimental treatments.

## Discussion

Many EDCs have been detected in estuarine waterways, including many commonly used pharmaceuticals and pesticides. Bifenthrin is a pyrethroid pesticide that enters watersheds primarily via runoff following application for urban and agricultural pest control (Kuivila et al., 2012). This compound is found in California surface waters at concentrations up to 106 ng/L (Weston & Lydy, 2012) and higher concentrations have been found in sediments throughout the world (Li et al., 2017). EE2 is a common component of oral contraceptives, which enters the waterways via wastewater effluent, at concentrations up to 7.0 ng/L (Adeel et al., 2017; King et al., 2016). While waste water effluents are primarily found in riverine environments, these rivers discharge into downstream estuarine and marine systems, introducing EE2 at low (ng/L) concentrations (Aris, Shamsuddin & Praveena, 2014). Environmentally relevant concentrations of these estrogenic EDCs have been shown to elicit adverse effects in fish including disruption of homeostasis, reproduction and development (Brander et al., 2016a; Brander et al., 2016b; DeCourten & Brander, 2017). Our results support previous findings illustrating the importance of testing for adverse effects of estrogenic EDCs at low doses, which can produce stronger effects than those observed at moderate doses (Brander et al., 2012; Vandenberg et al., 2012).

We found that the effects of EE2 exposure may be influenced by temperature in the F1 larvae, with the 28 °C EE2 treatment significantly altering expression of most genes tested. Many of the genes selected in this study are involved in the hypothalamic-pituitary-gonadal (HPG) axis and are useful in determining the effects of elevated temperature and EDC exposure on development and reproduction. We found that 28 °C EE2 treatment reduced the expression of the aromatase gene, CYP19b, which is primarily expressed in the brain and plays a pivotal role in sex determination by the conversion of androgens into estrogens (Diotel et al., 2010). Opposite of our findings with EE2, previous studies found that CYP19b expression was increased following exposure to environmental androgens or estrogens (Baumann et al., 2014; Filby et al., 2007). We found a similar pattern of expression in ESR2 and ESR3, which are nuclear, ligand-dependent estrogen receptors that can regulate estrogenic responses via direct mechanisms or by binding to estrogen response elements in the promoter regions of some genes (Filby & Tyler, 2005). Our findings in the 28 °C EE2 group are contrary to previous studies that reported an upregulation of estrogen receptors following exposure to estrogenic EDCs (Filby & Tyler, 2005; Filby et al., 2007; Rhee et al., 2011) indicating that these effects may be dependent on temperature. These discrepancies may also be attributed to the length of chemical exposure (0 hpf to 21 dph, including a 7–10 day embryonic incubation), and responses regulated via ligand–receptor feedback loops can be dependent on exposure period (Golshan et al., 2014). There is evidence that genes upregulated during earlier timepoints in early life stage exposure to EDCs can begin to down regulate as the exposure continues past 30 days post fertilization (Shved et al., 2008). Analogous to the expression of nuclear estrogen receptors, we found a reduction in the expression of GPR30, an estrogen receptor coupled with seven transmembrane G proteins that plays a pivotal role in reproductive success including activating oocyte maturation (Maggiolini & Picard, 2010; Pang, Dong & Thomas, 2008). The production of the enzymes 17β-HSD and 3β-HSD, which act as catalysts in the steroidogenesis pathway, is essential in the synthesis pathway of biologically active sex steroids (Marchais-Oberwinkler et al., 2011; Liu et al., 2012). We found that EE2 (28 °C) downregulated the expression of genes coding for the production of these enzymes, corroborating previous findings that production of this class of enzymes can be influenced by exposure to EE2 (Liu et al., 2012; Filby et al., 2007). Similarities between the expression of the above-mentioned genes indicate that the response to EE2 can exacerbated by temperature along the HPG axis following exposure at the larval stage. Changes in the expression of these genes may influence the sexual development and reproductive capacity of exposed fish. At the adult stage, F1 fish in this experiment were observed to have a more female biased sex ratio in the 28 °C EE2 than any other treatment (DeCourten & Brander, 2017). The observed changes in gene expression may explain the skewed sex ratios of the F1 adults in the 28 °C EE2 treatment (DeCourten & Brander, 2017) as sex of *M. beryllina* is determined in the larval stage (Conover & Fleisher, 1986; Strüssmann et al., 2010). Sex ratios deviating from the evolutionarily stable 50:50 males to females have the potential to result in population declines in *M. beryllina* (White et al., 2017).

We investigated the response of additional genes in larval tissue essential to reproductive function at later life stages. We found that the 28 °C EE2 treatment downregulated the production of GnRHR, which indicates that less gonadotropin releasing hormone (GnRH) is being produced in the hypothalamus (Levavi-Sivan et al., 2010). GnRH stimulates gonadotropin production in the gonads and is essential for gonad maturation, reproduction, and reproductive behaviors (Jodo, Ando & Urano, 2003). A similar pattern of expression was found with the FSHR gene indicating that EE2 (28 °C) downregulated the production of follicle stimulating hormone (FSH) in the pituitary of the larval fish. FSH levels can vary based on reproductive status of the individual and mediates steroidogenesis, gametogenesis and ovulation in the gonads (Rocha et al., 2009). A previous study using fish exposed to BPA, found an upregulation on GnRHR and FSHR, opposite of our finding (Rhee et al., 2011). However, Rhee et al. (2011) found similar expression patterns across GnRHR, FSHR, ERs, and CYP19a, indicating that these genes respond to EDCs similarly. Our findings align with Rhee et al. (2011) as the downregulation observed in the 28 °C EE2 treatment was similar across all genes we tested, indicating a similar response to EE2 exposure throughout the HPG axis. These results could explain the reduction in egg production by the F1 adults in the 28 °C EE2 treatment in this experiment as multiple genes within the HPG axis were downregulated within the 28 °C EE2 treatment, potentially impacting the development and reproduction (DeCourten & Brander, 2017). However, these changes in gene expression were not observed in the F1 adult gonad tissues, suggesting that gene expression altered immediately after EE2 exposure was not measurable later in life after rearing in clean water. Despite the lack of altered gene expression at the adult stage, we were able to measure changes to higher order processes (e.g., sex ratio, egg production) that could have been mediated through the expression of genes at the larval stage, which highlights the importance of testing across levels of biological organization (Brander, Hecht & Kuivila, 2015).

Previous literature has established that exposure to estrogenic EDCs has been shown to alter the expression of many genes involved in the reproductive and developmental pathways in gonadal tissues (Scholz & Gutzeit, 2000; Filby et al., 2007; Zhang et al., 2008). Likewise, increased temperature has been shown to influence expression of genes such as gonadal aromatase, which is the mechanism through which animals are able to achieve environmental sex determination (Kitano et al., 1999; Uchida et al., 2004). However, we were unable to detect any significant differences in the expression of genes in the parental ovaries (Fig. S1), parental testes (Fig. S2), F1 adult ovaries (Fig. S3), F1 adult testes (Fig. S4) in any treatments tested in this study. This is likely due to high variability of measurements between our samples and the loss of statistical power within our tests resulting from a high number of pairwise comparisons. These discrepancies with existing literature may also be explained by exposing our fish to EDCs at a salinity of 15 ppt because EE2 uptake can be influenced by changes in salinity (Blewett et al., 2013). Additionally, because we were only able to sample fish at one time point in each life stage we may have missed an opportunity to detect changes in gene expression that had occurred earlier on or there may have been unmeasured differences in protein or hormone concentrations that were not represented by gene expression quantifications at the end of the exposure. This may explain why these exposures resulted in phenotypic changes such as decreases in egg production within 48 h of the sampling time (DeCourten & Brander, 2017).

Like the genes involved in the HPG axis, we found that the IGF2 expression was reduced by exposure to EE2 at 28 °C. Production of IGFs are regulated by growth hormones, mediating the uptake of glucose and amino acids, and protein synthesis (Shamblott et al., 1995). A study with fathead minnows (*Pimephales promelas*) found that bifenthrin downregulated the expression of IGF2, when using higher concentrations than those used in our study (Beggel et al., 2011). This supports our findings that high doses or highly estrogenic EDCs, such as EE2 in our study, can downregulate the production of IGF2, although we did not find a significant difference between low concentrations of bifenthrin and the controls. Previous literature has shown that IGF2 transcription is not influenced by temperature (Gabillard et al., 2003), despite the growth of ectothermic organisms being heavily influenced by elevated temperatures (Moyano et al., 2016). Our results support the findings that temperature alone does not influence IGF2 transcription. However, we found that EE2 exposure, in combination with elevated temperature, had a greater effect on IGF2 regulation than either stressor alone.

It has been shown that temperature can exacerbate the feminizing effects of EE2 exposure and delay the development of zebrafish testes (Luzio et al., 2016). Previous studies have found that effects of estrogenic EDCs on molecular endpoints and development can be influenced by abiotic factors such as temperature and photoperiod (Brian et al., 2008; Jin et al., 2009). For example, Brian et al. (2008) found that the vitellogenin production increased at higher temperatures in fish exposed to a mixture of estrogenic EDCs. In the case of 17β-HSD, we found that increased temperature can also influence effects of bifenthrin exposure in the F2 generation. Bifenthrin has been shown to alter the expression of genes involved in hormone regulation producing higher order effects on reproduction (Brander et al., 2016b). It has been suggested that effects produced by concurrent exposure to EDCs and elevated temperature can negatively impact survival, development and reproduction, resulting in population declines over time (Brown et al., 2015).

Our results indicate that organisms exposed at early life stages are more susceptible to molecular effects of EDCs, as found with previous studies (Guillette Jr et al., 1995; Van Aerle et al., 2002), as we determined a greater effect of EDCs and temperature at the larval life stage than adult. In the case of FSHR expression, we found that the same pattern measured in the previous generation was also observed in their offspring. Parental exposure to environmental factors can result in phenotypic changes to offspring to allow for rapid response to environmental stressors (Burgess & Marshall, 2014). It has previously been shown that the effects of EDCs can be observed across multiple generations following exposure (Corrales et al., 2014). Transgenerational effects have been linked to lowered survival and reproduction in generations following parental exposure, including generations only exposed as germ cells (Schwindt et al., 2014; Bhandari, Vom Saal & Tillitt, 2015). However, similar effects between the F1 and F2 larvae were not seen in all genes with the expression of 17β-HSD and GPR30 differing between generations. For example, we saw a greater influence of bifenthrin on the expression of 17β-HSD and GRP30 in the F2 larvae, which were exposed indirectly as germ cells, than the previous generation. Our previous findings suggest that EDCs can have more deleterious effects on development in indirectly exposed larvae (DeCourten & Brander, 2017). Our results indicate that the full impact of some EDCs may not be realized when conducting exposures on a single generation. Multigenerational testing is important to determine the effects of parental exposure and potential epigenetic modifications caused by EDC exposure that may result in persistent effects to exposed populations (Brander, Biales & Connon, 2017).

## Conclusions

We found that increases in temperature can increase the effects of EDC exposure, as we observed more alterations of gene expression in higher temperatures. Based off predictions of temperature increases over the next 100 years by the IPCC 2013 report (Stocker et al., 2013), we can expect to see rising temperatures influence how organisms respond to exposure to EDCs. Our findings confirm the importance for research incorporating multiple stressors to assess the risk of anthropogenic stressors to aquatic organisms. We found that the changes in larval gene expression resulting from exposure to a combination of EDCs and temperature can result in higher order effects that are measurable later in life after molecular endpoints are no longer affected. These findings imply that organisms exposed briefly as larvae can be affected by these stressors throughout their lives regardless of time allotted for recovery. Additionally, we found that effects of EDCs can be carried over to the next generation or in some cases produce different effects in subsequent generations. These findings suggest mechanisms dictating generational transfer of effects following parental exposure may not affect all molecular endpoints equally. Further research is needed to determine the effects of parental exposure on subsequent generations. We found that larval life stages were more sensitive to EDC exposure than adults, and that changes in gene expression following EDC exposure can be further influenced by temperature. Our findings will inform the long-term management of aquatic systems vulnerable to pollution under future climate change scenarios, many of which provide critical habitat for fishes during early life stages.

##  Supplemental Information

10.7717/peerj.6156/supp-1 Table S1 Sample sizes for qPCR analyses The number of male (M) and female (F) adult gonads, or number of larvae used in analysis of gene expression within each replicate. Number of replicates (n) is indicated for each treatment

10.7717/peerj.6156/supp-2 Table S2 *P*-values from pairwise comparisons for larval qPCR results P values produced by Tukey’s post hoc comparisons following a two-way ANOVA for each gene tested in the F1 and F2 larvae. Significance is denoted by bolded text at the *p* < 0.05 (*), *p* < 0.01(**), and *P* < 0.001(***) levels. Within the F1 larvae *n* = 5 for all treatments. Within the F2 *n* = 5 for all treatments except 28 °C bifenthrin and 28 °C MeOH (*n* = 4).

10.7717/peerj.6156/supp-3 Figure S1 Relative expression of genes in parental ovaries

10.7717/peerj.6156/supp-4 Figure S2 Relative gene expression of parental testes

10.7717/peerj.6156/supp-5 Figure S3 Relative gene expression in F1 ovaries

10.7717/peerj.6156/supp-6 Figure S4 Relative expression of genes in F1 testes

10.7717/peerj.6156/supp-7 File S1 R code used for statistical analyses Example of code used to analyze data, accounting for pseudoreplicate samples.

10.7717/peerj.6156/supp-8 Data S1 Normalized fold change (log2) values for each data set used in this study Fold change (log2) values are provided for each data set in this study. Please refer to the description tab for more information on calculations used to normalize CT values and convert to fold-change values

## Additional Information and Declarations

### Data Availability

The following information was supplied regarding data availability: The raw data is provided in the Supplementary Files.
