# Supplementary material for: Direct and indirect parental exposure to endocrine disruptors and elevated temperature influences gene expression across generations in a euryhaline model fish
Source: PeerJ. 2019 Jan 8;7:e6156. doi: 10.7717/peerj.6156 (PMC6329337; doi:10.7717/peerj.6156)
Supplement: Table S1 — The number of male (M) and female (F) adult gonads, or number of larvae used in analysis of gene expression within each replicate. Number of replicates (n) is indicated for each treatment [file peerj-07-6156-s001.docx]

| **Generation** | **Treatment** | **Replicate** | **Number**  **of samples** | **Treatment** | **Replicate** | **Number of fish** | **Treatment** | **Replicate** | **Number of samples** |
| --- | --- | --- | --- | --- | --- | --- | --- | --- | --- |
| F0 | BF (22^o^C) | 1 | 2F, 1M | EE2 (22^o^C) | 1 | 2F, 2M | Control (22^o^C) | 1 | 2F, 3M |
| Adults | n=5 | 2 | 1F, 2M | n=5 | 2 | 2F, 2M | n=5 | 2 | 1F, 2M |
|  |  | 3 | 2F, 1M |  | 3 | 2F, 2M |  | 3 | 2F, 1M |
|  |  | 4 | 2F, 1M |  | 4 | 1F, 2M |  | 4 | 1F, 1M |
|  |  | 5 | 3F, 1M |  | 5 | 1F, 2M |  | 5 | 1F, 3M |
|  | BF (28^o^C) | 1 | 3F, 1M | EE2 (28^o^C) | 1 | 2F, 2M | Control (28^o^C) | 1 | 2F, 1M |
|  | n=5 | 2 | 2F, 2M | n=5 | 2 | 2F, 1M | n=5 | 2 | 2F, 2M |
|  |  | 3 | 2F, 2M |  | 3 | 2F, 1M |  | 3 | 1F, 2M |
|  |  | 4 | 2F, 1M |  | 4 | 1F, 2M |  | 4 | 1F, 1M |
|  |  | 5 | 2F, 1M |  | 5 | 2F, 2M |  | 5 | 1F, 2M |
| F1 | BF (22^o^C) | 1 | 2F, 1M | EE2 (22^o^C) | 1 | 3F, 2M | Control (22^o^C) | 1 | 1F,1M |
| Adults | n=5 | 2 | 2F, 1M | n=5 | 2 | 2F, 1M | n=5 | 2 | 2F, 2M |
|  |  | 3 | 2F, 2M |  | 3 | 3F, 3M |  | 3 | 3F, 1M |
|  |  | 4 | 2F, 1M |  | 4 | 2F, 1M |  | 4 | 2F, 1M |
|  |  | 5 | 2F, 1M |  | 5 | 3F, 2M |  | 5 | 2F, 1M |
|  | BF (28^o^C) | 1 | 2F, 2M | EE2 (28^o^C) | 1 | 3F, 1M | Control (28^o^C) | 1 | 1F, 2M |
|  | n=5 | 2 | 2F, 1M | n=4 | 2 | 2F, 3M | n=4 | 2 | 3F, 2M |
|  |  | 3 | 1F, 1M |  | 4 | 2F, 0M |  | 4 | 2F, 2M |
|  |  | 4 | 2F, 1M |  | 5 | 3F, 2M |  | 5 | 2F, 1M |
|  |  | 5 | 1F, 2M |  |  |  |  |  |  |
| F1 | BF (22^o^C) | 1 | 2 | EE2 (22^o^C) | 1 | 2 | Control (22^o^C) | 1 | 2 |
| Larvae | n=5 | 2 | 1 | n=5 | 2 | 2 | n=5 | 2 | 1 |
|  |  | 3 | 3 |  | 3 | 1 |  | 3 | 2 |
|  |  | 4 | 2 |  | 4 | 3 |  | 4 | 2 |
|  |  | 5 | 2 |  | 5 | 2 |  | 5 | 2 |
|  | BF (28^o^C) | 1 | 2 | EE2 (28^o^C) | 1 | 1 | Control (28^o^C) | 1 | 2 |
|  | n=5 | 2 | 2 | n=5 | 2 | 2 | n=5 | 2 | 2 |
|  |  | 3 | 2 |  | 3 | 2 |  | 3 | 2 |
|  |  | 4 | 1 |  | 4 | 2 |  | 4 | 2 |
|  |  | 5 | 2 |  | 5 | 3 |  | 5 | 2 |
| F2 | BF (22^o^C) | 2 | 2 | EE2 (22^o^C) | 1 | 1 | Control (22^o^C) | 1 | 2 |
| Larvae | n=4 | 3 | 2 | n=5 | 2 | 2 | n=5 | 2 | 3 |
|  |  | 4 | 4 |  | 3 | 2 |  | 3 | 1 |
|  |  | 5 | 1 |  | 4 | 2 |  | 4 | 2 |
|  |  |  |  |  | 5 | 2 |  | 5 | 3 |
|  | BF (28^o^C) | 1 | 2 | EE2 (28^o^C) | 1 | 2 | Control (28^o^C) | 1 | 1 |
|  | n=4 | 2 | 1 | n=3 | 2 | 2 | n=4 | 2 | 1 |
|  |  | 4 | 1 |  | 3 | 2 |  | 3 | 2 |
|  |  | 5 | 2 |  |  |  |  | 4 | 2 |
|  |  |  |  |  |  |  |  |  |  |
|  |  |  |  |  |  |  |  |  |  |
