# Supplementary material for: Direct and indirect parental exposure to endocrine disruptors and elevated temperature influences gene expression across generations in a euryhaline model fish
Source: PeerJ. 2019 Jan 8;7:e6156. doi: 10.7717/peerj.6156 (PMC6329337; doi:10.7717/peerj.6156)
Supplement: Table S2 — P values produced by Tukey’s post hoc comparisons following a two-way ANOVA for each gene tested in the F1 and F2 larvae. Significance is denoted by bolded text at the p < 0.05 (*), p < 0.01(**), and P < 0.001(***) levels. Within the F1 larvae n = 5 for all treatments. Within the F2 n = 5 for all treatments except 28 °C bifenthrin and 28 °C MeOH (n = 4). [file peerj-07-6156-s002.docx]

| Gene | Comparison | P value | P value |
| --- | --- | --- | --- |

| ESR1 |  | F1 Larvae | F2 Larvae |
| --- | --- | --- | --- |
|  | 22°C MeOH – 28°C MeOH | 1.00000 | 0.987 |
|  | 22°C MeOH – 28° Bif | 0.9041 | 0.941 |
|  | 22°C MeOH – 28° EE2 | 0.1537 | 0.991 |
|  | 22°C MeOH – 22° Bif | 1.00000 | 0.923 |
|  | 22°C MeOH – 22°EE2 | 1.00000 | 0.832 |
|  | 22°C EE2 – 28°C EE2 | 0.0627 | 0.997 |
|  | 22°C EE2 – 28°C MeOH | 1.00000 | 0.996 |
|  | 22°C EE2 – 28°C Bif | 0.8001 | 1.000 |
|  | 22°C EE2 – 22°C Bif | 1.00000 | 1.000 |
|  | 22°C Bif – 28°C Bif | 0.8547 | 1.000 |
|  | 22°C Bif – 28°C EE2 | 0.0952 | 1.000 |
|  | 22°C Bif – 28°C MeOH | 1.00000 | 1.000 |
|  | 28° MeOH – 28°C Bif | 0.8815 | 1.000 |
|  | 28° MeOH – 28°C EE2 | 0.1378 | 1.000 |
|  | 28°C EE2 – 28°C Bif | 0.4973 | 1.000 |
| ESR2 | 22°C MeOH – 28°C MeOH | 1.0000 | 0.998 |
|  | 22°C MeOH – 28° Bif | 0.7870 | 0.973 |
|  | 22°C MeOH – 28° EE2 | **0.0268*** | 0.998 |
|  | 22°C MeOH – 22° Bif | 1.0000 | 0.999 |
|  | 22°C MeOH – 22°EE2 | 1.0000 | 0.888 |
|  | 22°C EE2 – 28°C EE2 | **0.0268*** | 0.996 |
|  | 22°C EE2 – 28°C MeOH | 1.0000 | 0.992 |
|  | 22°C EE2 – 28°C Bif | 0.7884 | 1.000 |
|  | 22°C EE2 – 22°C Bif | 1.0000 | 0.985 |
|  | 22°C Bif – 28°C Bif | 0.7665 | 0.999 |
|  | 22°C Bif – 28°C EE2 | **0.0245*** | 1.000 |
|  | 22°C Bif – 28°C MeOH | 1.0000 | 1.000 |
|  | 28° MeOH – 28°C Bif | 0.7735 | 1.000 |
|  | 28° MeOH – 28°C EE2 | **0.0252*** | 1.000 |
|  | 28°C EE2 – 28°C Bif | 0.3272 | 1.000 |
| ESR3 | 22°C MeOH – 28°C MeOH | 1.00000 | 0.999 |
|  | 22°C MeOH – 28° Bif | 0.4281 | 0.999 |
|  | 22°C MeOH – 28° EE2 | **0.0362*** | 0.999 |
|  | 22°C MeOH – 22° Bif | 1.00000 | 1.000 |
|  | 22°C MeOH – 22°EE2 | 1.00000 | 0.996 |
|  | 22°C EE2 – 28°C EE2 | **0.0425*** | 1.000 |
|  | 22°C EE2 – 28°C MeOH | 1.00000 | 1.000 |
|  | 22°C EE2 – 28°C Bif | 0.4648 | 1.000 |
|  | 22°C EE2 – 22°C Bif | 1.00000 | 1.000 |
|  | 22°C Bif – 28°C Bif | 0.3910 | 1.000 |
|  | 22°C Bif – 28°C EE2 | **0.0307*** | 1.000 |
|  | 22°C Bif – 28°C MeOH | 1.00000 | 1.000 |
|  | 28° MeOH – 28°C Bif | 0.4178 | 1.000 |
|  | 28° MeOH – 28°C EE2 | **0.0347*** | 1.000 |
|  | 28°C EE2 – 28°C Bif | 0.9346 | 1.000 |
| GPR30 | 22°C MeOH – 28°C MeOH | 1.0000 | 0.51013 |
|  | 22°C MeOH – 28° Bif | 0.7675 | 0.69454 |
|  | 22°C MeOH – 28° EE2 | **0.0261*** | 0.70695 |
|  | 22°C MeOH – 22° Bif | 1.0000 | **0.00851**** |
|  | 22°C MeOH – 22°EE2 | 1.0000 | **0.00294**** |
|  | 22°C EE2 – 28°C EE2 | **0.0226*** | **<0.001***** |
|  | 22°C EE2 – 28°C MeOH | 1.0000 | **<0.001***** |
|  | 22°C EE2 – 28°C Bif | 0.7318 | **<0.001***** |
|  | 22°C EE2 – 22°C Bif | 1.0000 | 0.99678 |
|  | 22°C Bif – 28°C Bif | 0.7911 | **<0.001***** |
|  | 22°C Bif – 28°C EE2 | **0.0376*** | **<0.001***** |
|  | 22°C Bif – 28°C MeOH | 1.0000 | **<0.001***** |
|  | 28° MeOH – 28°C Bif | 0.7549 | 0.99962 |
|  | 28° MeOH – 28°C EE2 | **0.0248*** | 0.99997 |
|  | 28°C EE2 – 28°C Bif | 0.3351 | 1.000000 |
| GnRHR | 22°C MeOH – 28°C MeOH | 1.00000 | 0.999 |
|  | 22°C MeOH – 28° Bif | 0.6884 | 0.986 |
|  | 22°C MeOH – 28° EE2 | **0.0356*** | 1.000 |
|  | 22°C MeOH – 22° Bif | 1.00000 | 0.999 |
|  | 22°C MeOH – 22°EE2 | 1.00000 | 0.969 |
|  | 22°C EE2 – 28°C EE2 | **0.0365*** | 0.996 |
|  | 22°C EE2 – 28°C MeOH | 1.00000 | 0.998 |
|  | 22°C EE2 – 28°C Bif | 0.6952 | 1.000 |
|  | 22°C EE2 – 22°C Bif | 1.00000 | 0.999 |
|  | 22°C Bif – 28°C Bif | 0.6977 | 1.000 |
|  | 22°C Bif – 28°C EE2 | **0.0369*** | 1.000 |
|  | 22°C Bif – 28°C MeOH | 1.00000 | 1.000 |
|  | 28° MeOH – 28°C Bif | 0.7145 | 1.000 |
|  | 28° MeOH – 28°C EE2 | **0.0393*** | 1.000 |
|  | 28°C EE2 – 28°C Bif | 0.4947 | 0.999 |
| IGF2 | 22°C MeOH – 28°C MeOH | 1.00000 | 1.000 |
|  | 22°C MeOH – 28° Bif | 0.7359 | 0.999 |
|  | 22°C MeOH – 28° EE2 | **0.0236*** | 0.999 |
|  | 22°C MeOH – 22° Bif | 1.00000 | 0.999 |
|  | 22°C MeOH – 22°EE2 | 1.00000 | 0.972 |
|  | 22°C EE2 – 28°C EE2 | **0.0229*** | 1.000 |
|  | 22°C EE2 – 28°C MeOH | 1.00000 | 0.998 |
|  | 22°C EE2 – 28°C Bif | 0.7265 | 0.999 |
|  | 22°C EE2 – 22°C Bif | 1.00000 | 0.999 |
|  | 22°C Bif – 28°C Bif | 0.7381 | 1.000 |
|  | 22°C Bif – 28°C EE2 | **0.0240*** | 1.000 |
|  | 22°C Bif – 28°C MeOH | 1.00000 | 1.000 |
|  | 28° MeOH – 28°C Bif | 0.7515 | 1.000 |
|  | 28° MeOH – 28°C EE2 | **0.0252*** | 1.000 |
|  | 28°C EE2 – 28°C Bif | 0.3482 | 1.000 |
| CYP19b | 22°C MeOH – 28°C MeOH | 0.10000 | 1.000 |
|  | 22°C MeOH – 28° Bif | 0.8133 | 1.000 |
|  | 22°C MeOH – 28° EE2 | **0.0297*** | 0.990 |
|  | 22°C MeOH – 22° Bif | 1.000000 | 1.000 |
|  | 22°C MeOH – 22°EE2 | 1.000000 | 0.900 |
|  | 22°C EE2 – 28°C EE2 | **0.0195*** | 0.992 |
|  | 22°C EE2 – 28°C MeOH | 1.000000 | 0.979 |
|  | 22°C EE2 – 28°C Bif | 0.7544 | 0.999 |
|  | 22°C EE2 – 22°C Bif | 1.00000 | 0.884 |
|  | 22°C Bif – 28°C Bif | 0.7563 | 0.984 |
|  | 22°C Bif – 28°C EE2 | **0.0231*** | 0.998 |
|  | 22°C Bif – 28°C MeOH | 1.00000 | 0.999 |
|  | 28° MeOH – 28°C Bif | 0.7544 | 1.000 |
|  | 28° MeOH – 28°C EE2 | **0.0231*** | 1.000 |
|  | 28°C EE2 – 28°C Bif | 0.3252 | 1.000 |
| FSHR | 22°C MeOH – 28°C MeOH | 0.99968 | 0.62813 |
|  | 22°C MeOH – 28° Bif | 0.60688 | 0.69645 |
|  | 22°C MeOH – 28° EE2 | **0.01362*** | **0.00156**** |
|  | 22°C MeOH – 22° Bif | 1.000000 | 0.79774 |
|  | 22°C MeOH – 22°EE2 | 1.000000 | 0.59192 |
|  | 22°C EE2 – 28°C EE2 | **0.01465*** | **<0.001***** |
|  | 22°C EE2 – 28°C MeOH | 0.99947 | 1.000 |
|  | 22°C EE2 – 28°C Bif | 0.62506 | 1.000 |
|  | 22°C EE2 – 22°C Bif | 1.000000 | 0.99963 |
|  | 22°C Bif – 28°C Bif | 0.63697 | 0.99996 |
|  | 22°C Bif – 28°C EE2 | **0.01534*** | **<0.001***** |
|  | 22°C Bif – 28°C MeOH | 0.99928 | 0.99963 |
|  | 28° MeOH – 28°C Bif | 0.44192 | 1.000 |
|  | 28° MeOH – 28°C EE2 | **0.00694*** | **<0.001***** |
|  | 28°C EE2 – 28°C Bif | 0.044059 | **<0.001***** |
| 17β-HSD | 22°C MeOH – 28°C MeOH | 1.00000 | 0.9909 |
|  | 22°C MeOH – 28° Bif | 0.7240 | **0.0249*** |
|  | 22°C MeOH – 28° EE2 | **0.0258*** | 0.8633 |
|  | 22°C MeOH – 22° Bif | 1.00000 | 0.9943 |
|  | 22°C MeOH – 22°EE2 | 1.00000 | 0.9760 |
|  | 22°C EE2 – 28°C EE2 | **0.0229*** | 0.9956 |
|  | 22°C EE2 – 28°C MeOH | 1.00000 | 1.00000 |
|  | 22°C EE2 – 28°C Bif | 06931 | 0.0862 |
|  | 22°C EE2 – 22°C Bif | 1.00000 | 1.0000 |
|  | 22°C Bif – 28°C Bif | 0.7620 | 0.0845 |
|  | 22°C Bif – 28°C EE2 | **0.0300*** | 0.9884 |
|  | 22°C Bif – 28°C MeOH | 1.00000 | 1.0000 |
|  | 28° MeOH – 28°C Bif | 0.7520 | 0.0924 |
|  | 28° MeOH – 28°C EE2 | **0.0289*** | 0.9920 |
|  | 28°C EE2 – 28°C Bif | 0.3781 | 0.3095 |
| 3β-HSD | 22°C MeOH – 28°C MeOH | 1.00000 | 0.153 |
|  | 22°C MeOH – 28° Bif | 0.7290 | 0.293 |
|  | 22°C MeOH – 28° EE2 | **0.0264*** | 0.304 |
|  | 22°C MeOH – 22° Bif | 1.00000 | 1.000 |
|  | 22°C MeOH – 22°EE2 | 1.00000 | 0.506 |
|  | 22°C EE2 – 28°C EE2 | **0.0273*** | 0.988 |
|  | 22°C EE2 – 28°C MeOH | 1.00000 | 0.939 |
|  | 22°C EE2 – 28°C Bif | 0.7368 | 0.995 |
|  | 22°C EE2 – 22°C Bif | 1.00000 | 0.693 |
|  | 22°C Bif – 28°C Bif | 0.7334 | 0.465 |
|  | 22°C Bif – 28°C EE2 | **0.0268*** | 0.456 |
|  | 22°C Bif – 28°C MeOH | 1.00000 | 0.288 |
|  | 28° MeOH – 28°C Bif | 0.7448 | 0.999 |
|  | 28° MeOH – 28°C EE2 | **0.0281*** | 1.000 |
|  | 28°C EE2 – 28°C Bif | 0.3792 | 1.000 |
