# Supplementary figures and images for: Direct and indirect parental exposure to endocrine disruptors and elevated temperature influences gene expression across generations in a euryhaline model fish
Source: PeerJ. 2019 Jan 8;7:e6156. doi: 10.7717/peerj.6156 (PMC6329337; doi:10.7717/peerj.6156)

Relative gene expression of parental ovaries

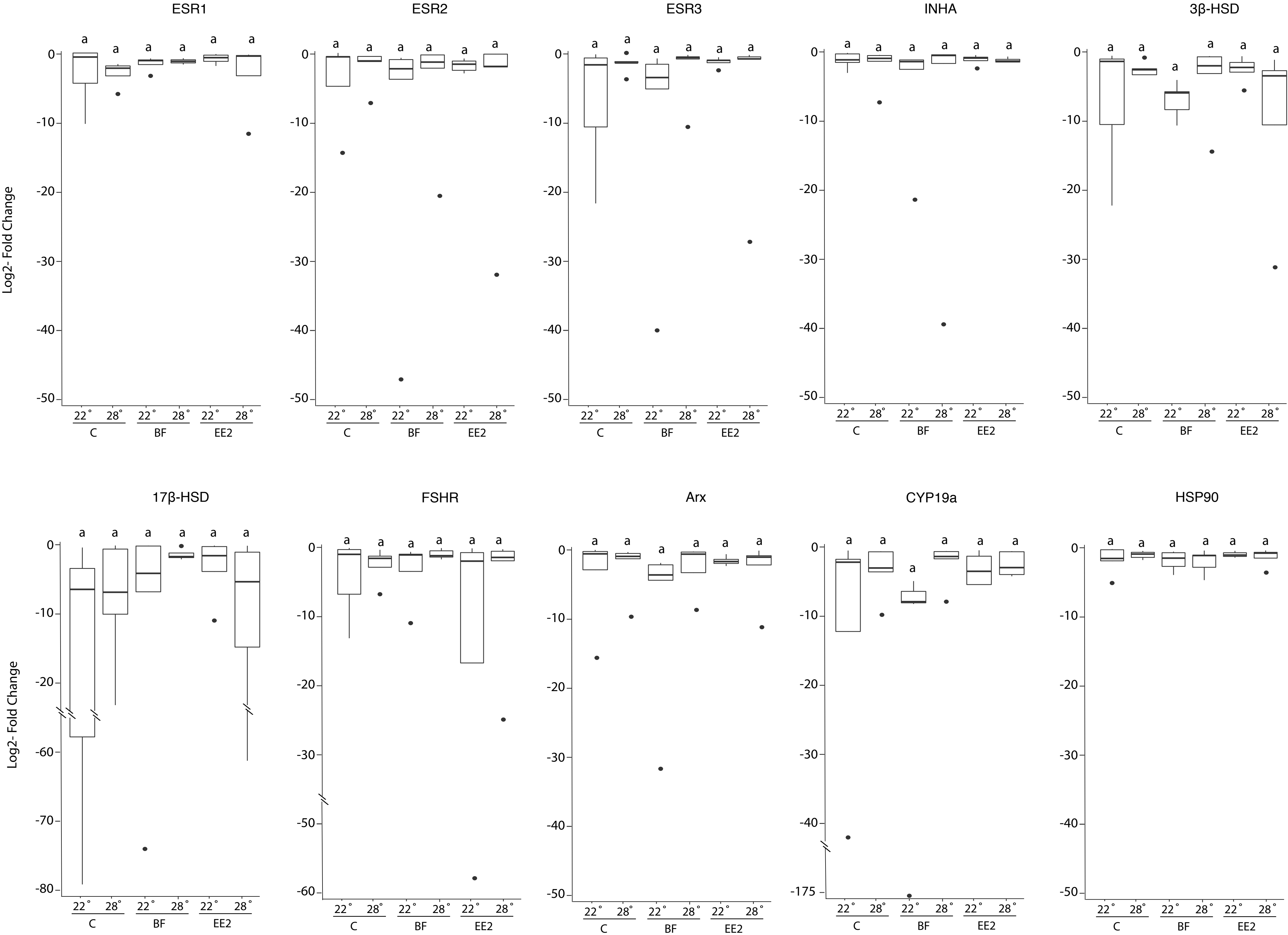

Supplement: Figure S1 [file peerj-07-6156-s003.pdf]

Relative gene expression of parental testes

ESR1

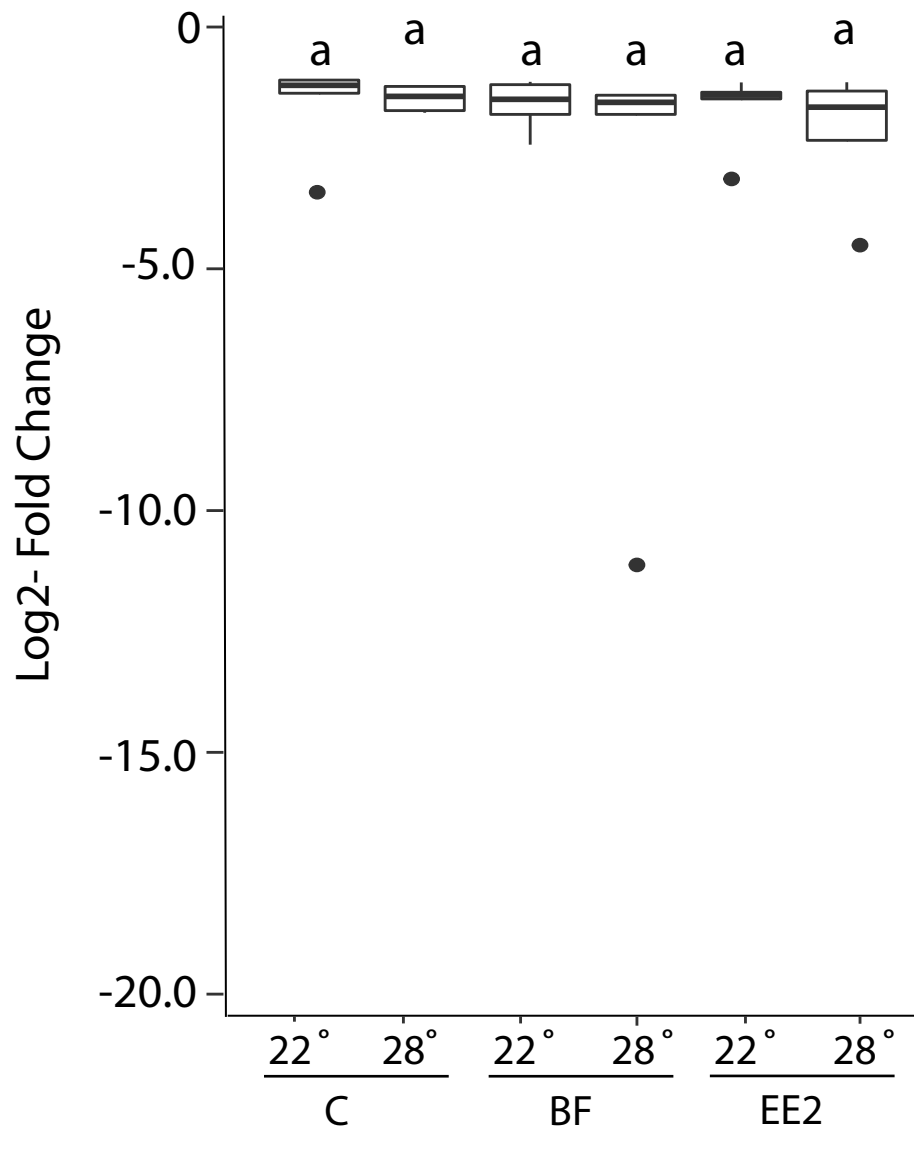

ESR2

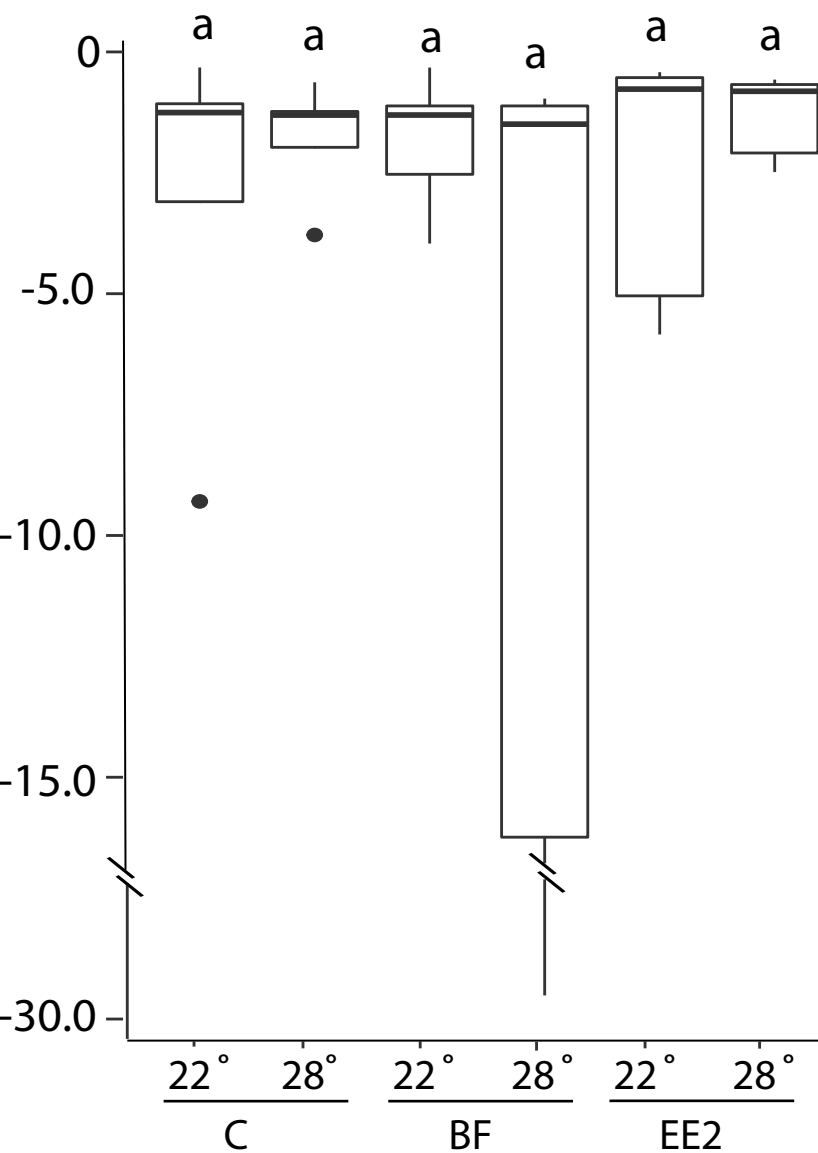

ESR3

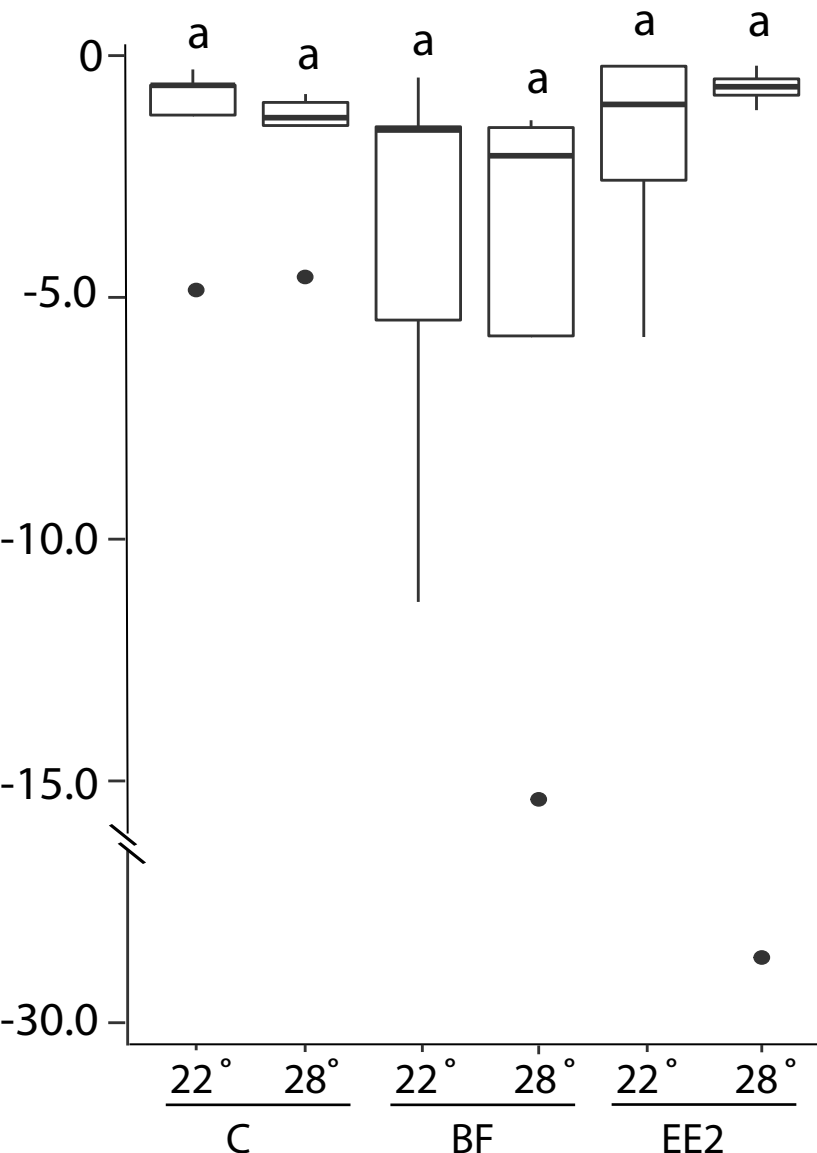

INHA

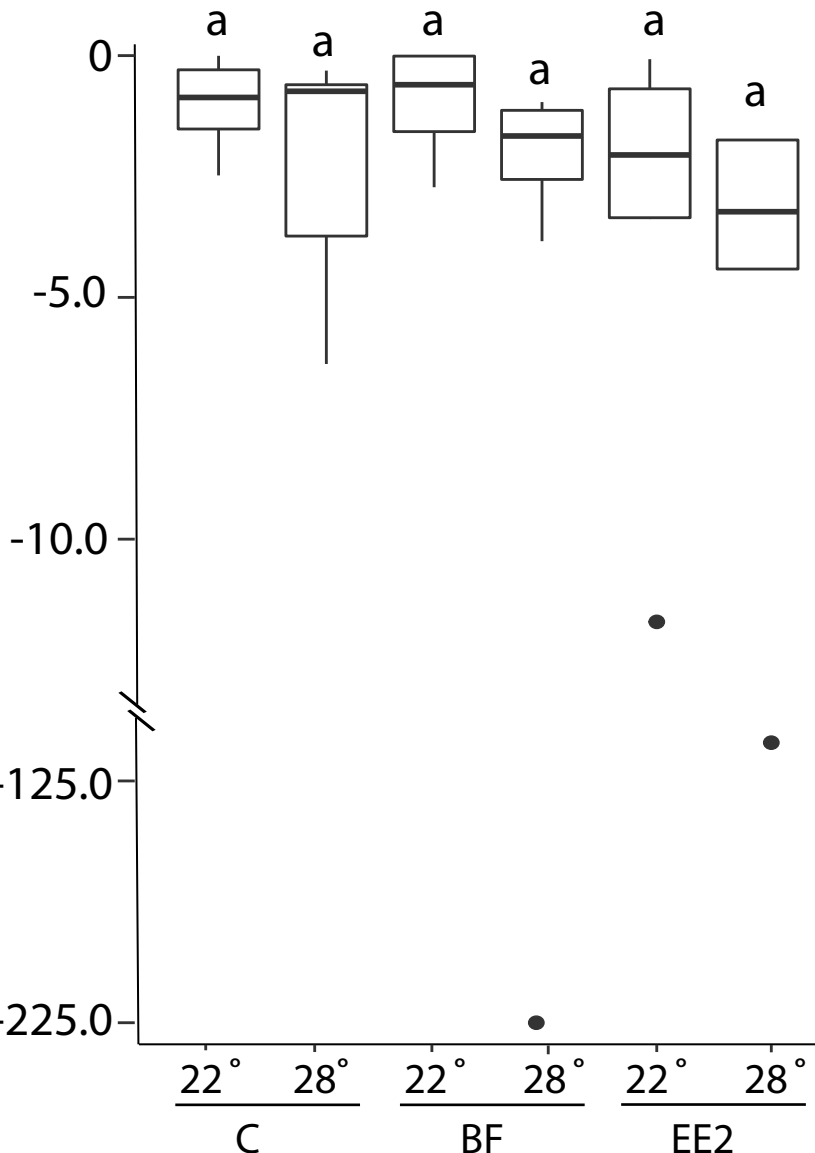

3β-HSD

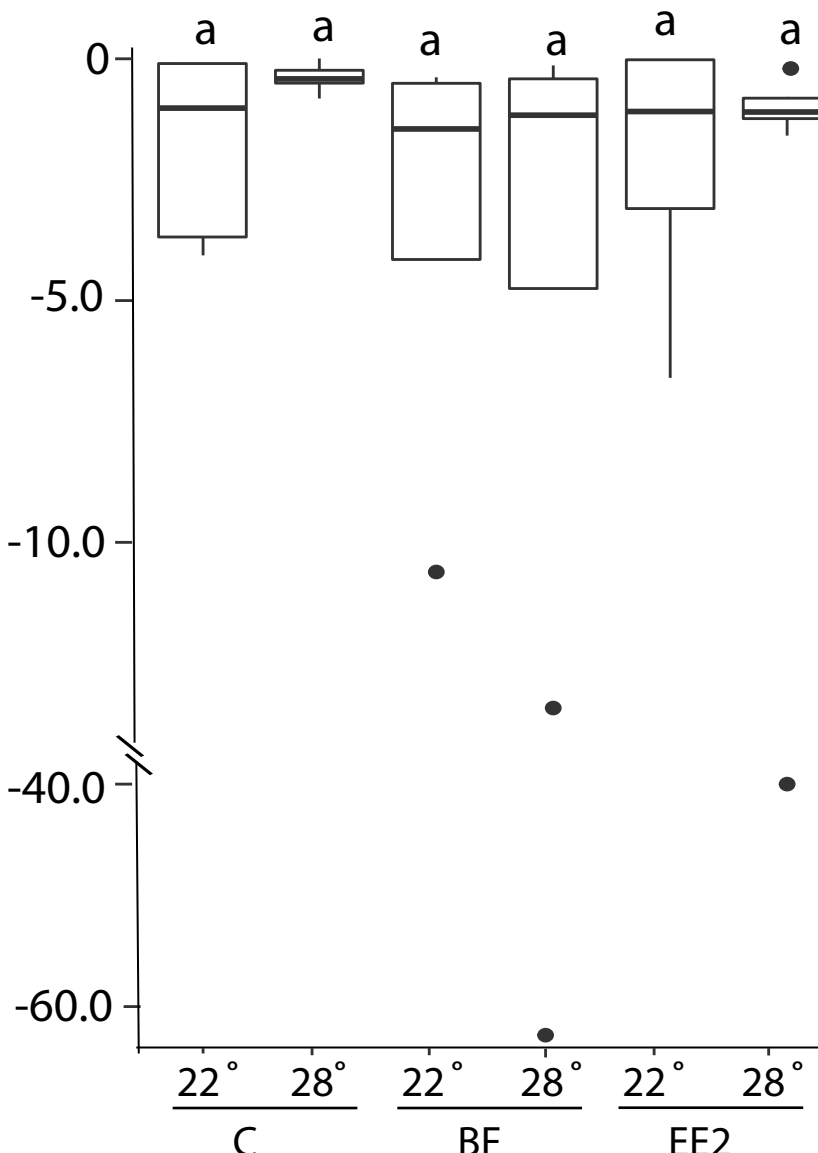

17β-HSD

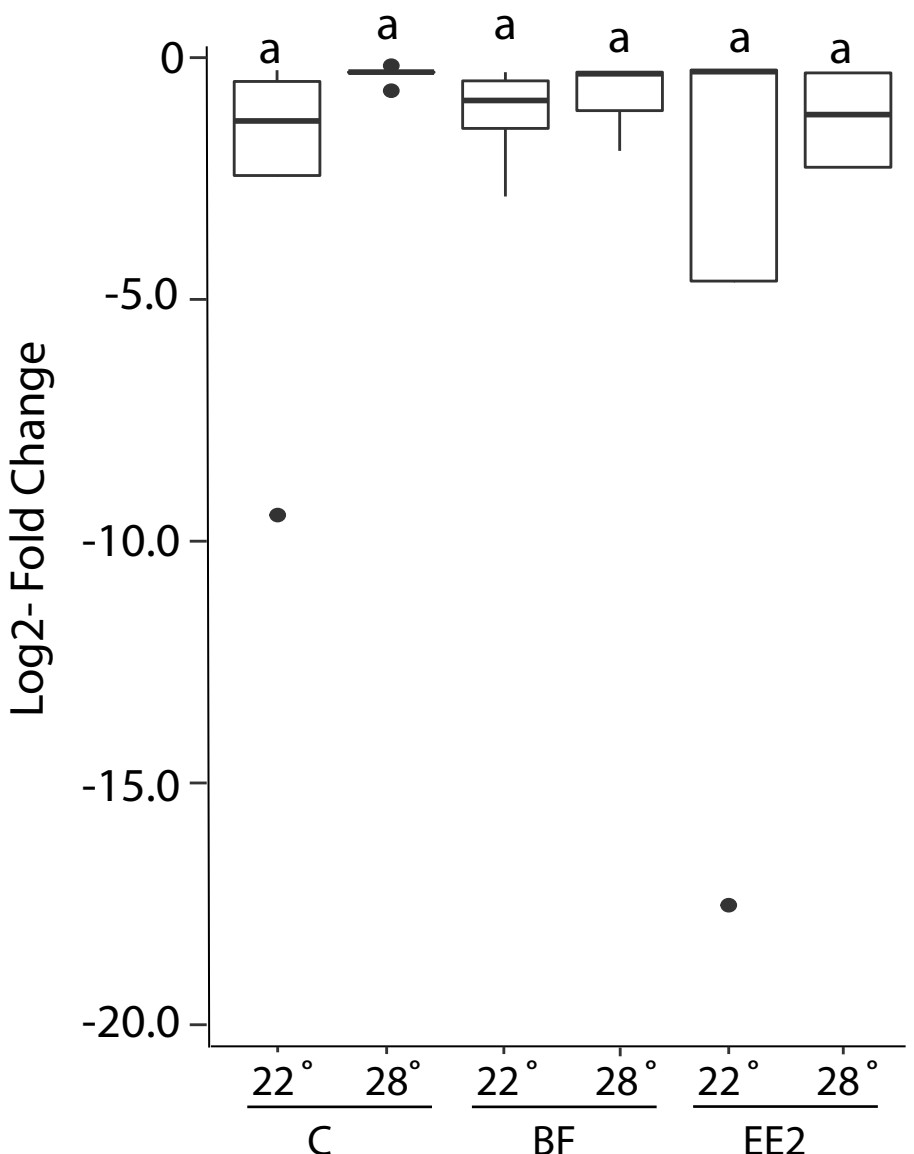

FSHR

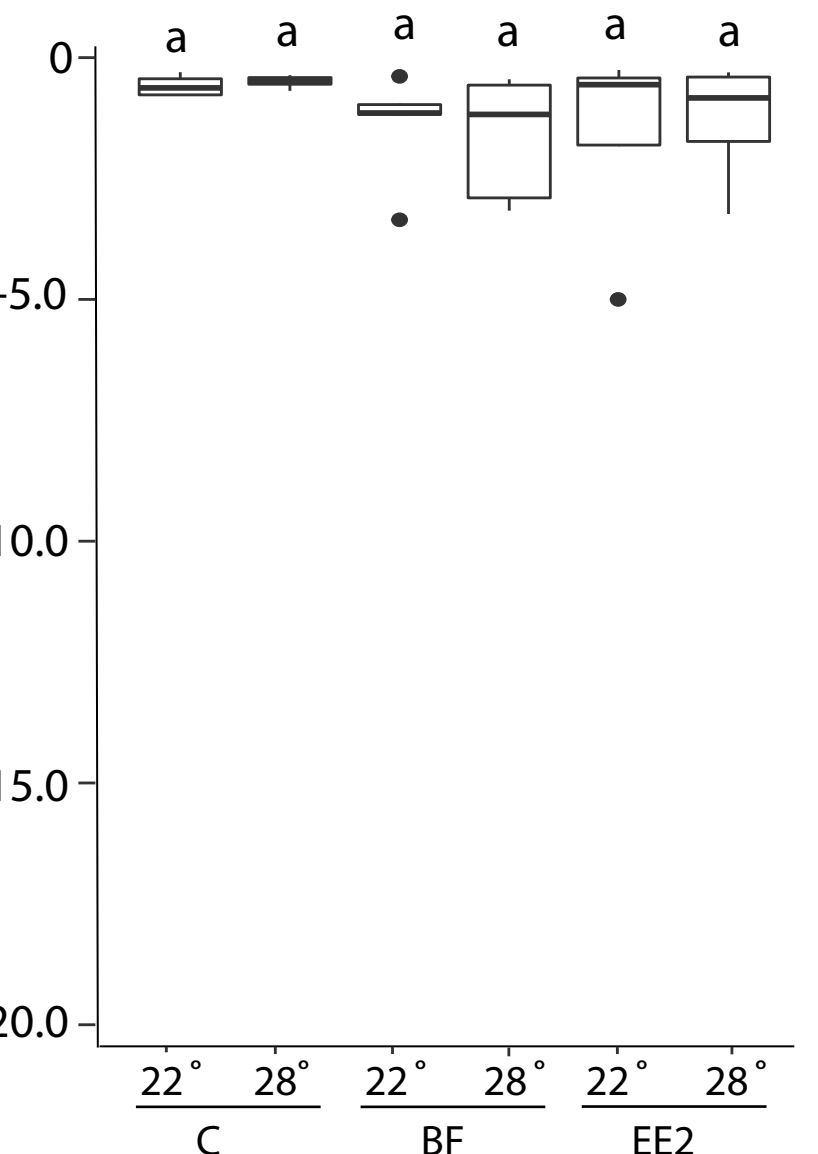

Arx

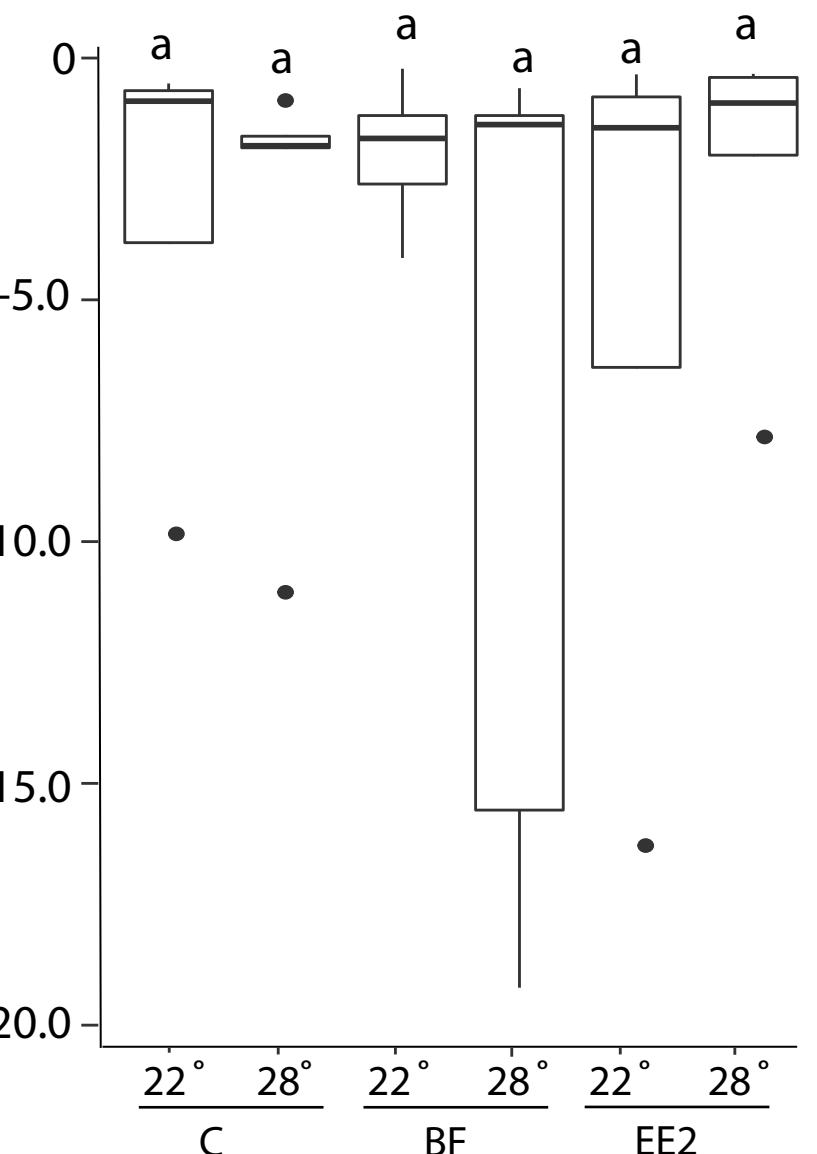

CYP19a

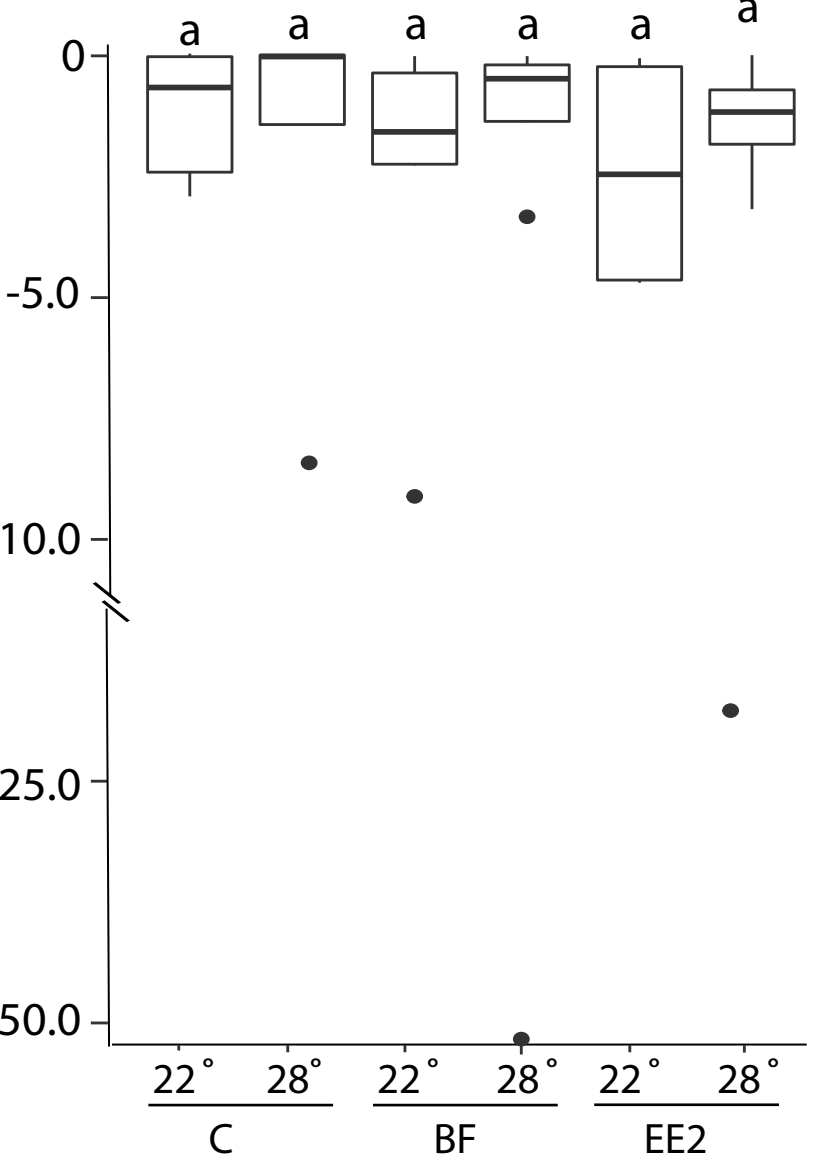

HSP90

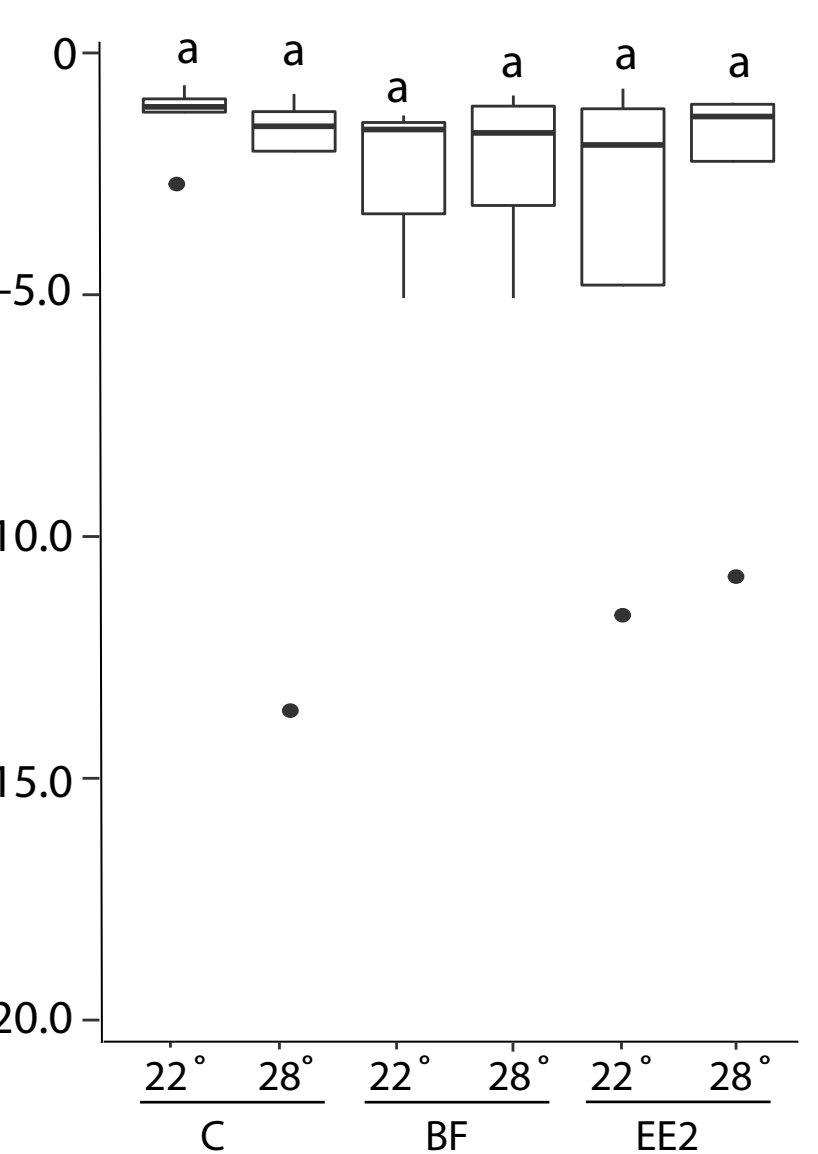

Supplement: Figure S2 [file peerj-07-6156-s004.pdf]

Relative gene expression of F1 ovaries

ESR1

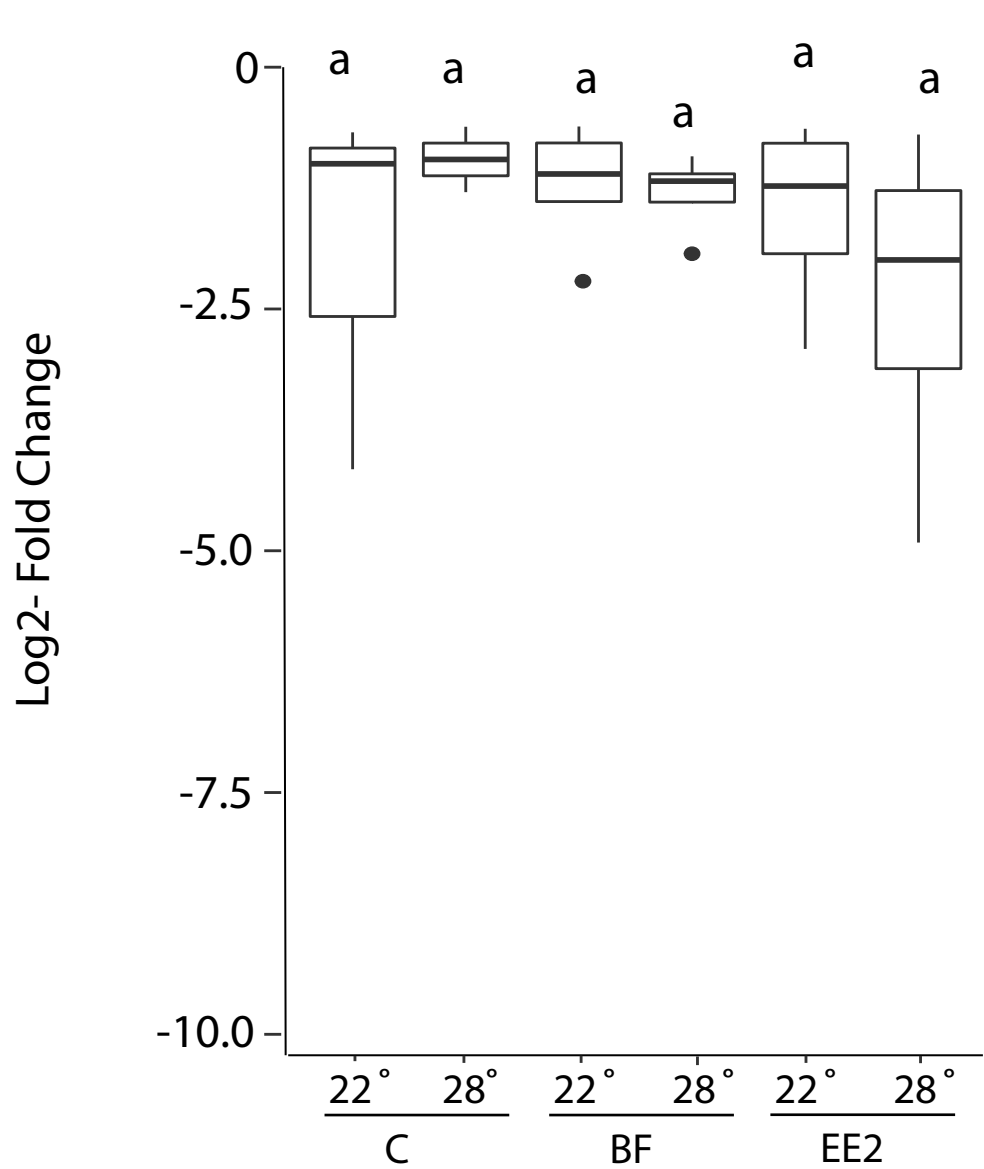

ESR2

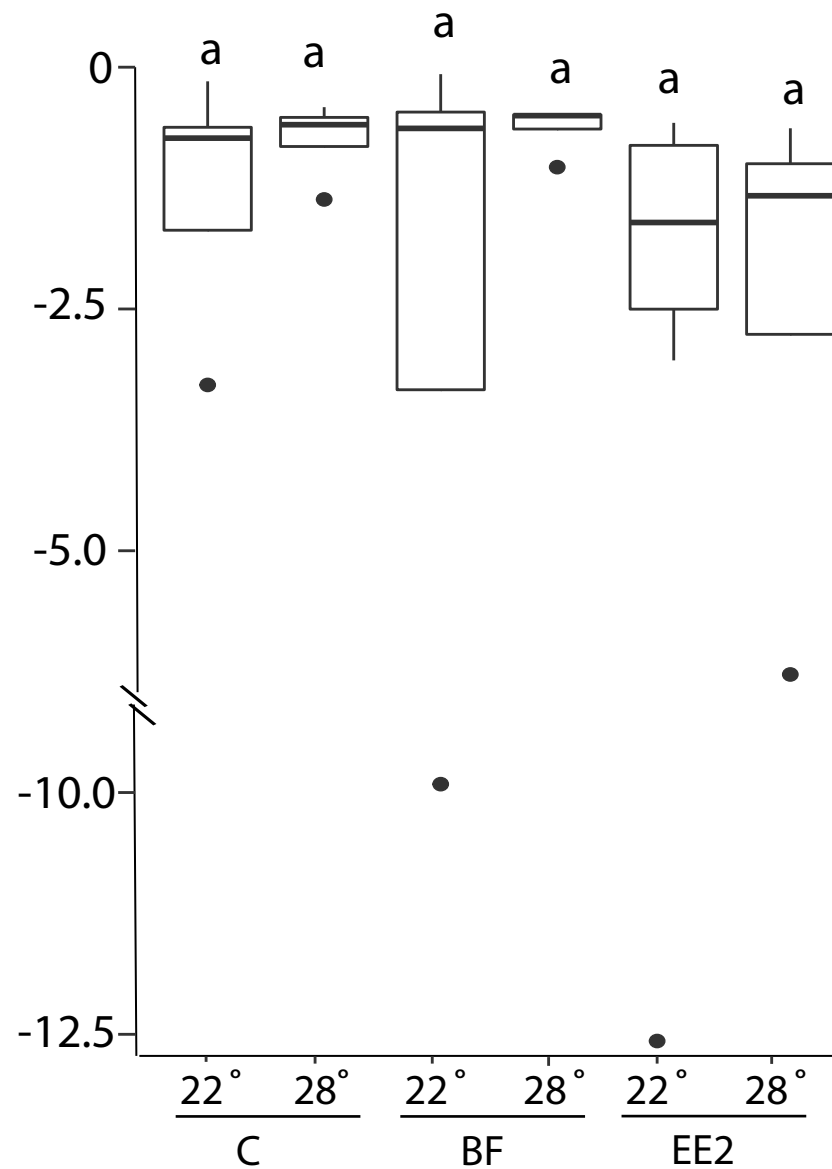

ESR3

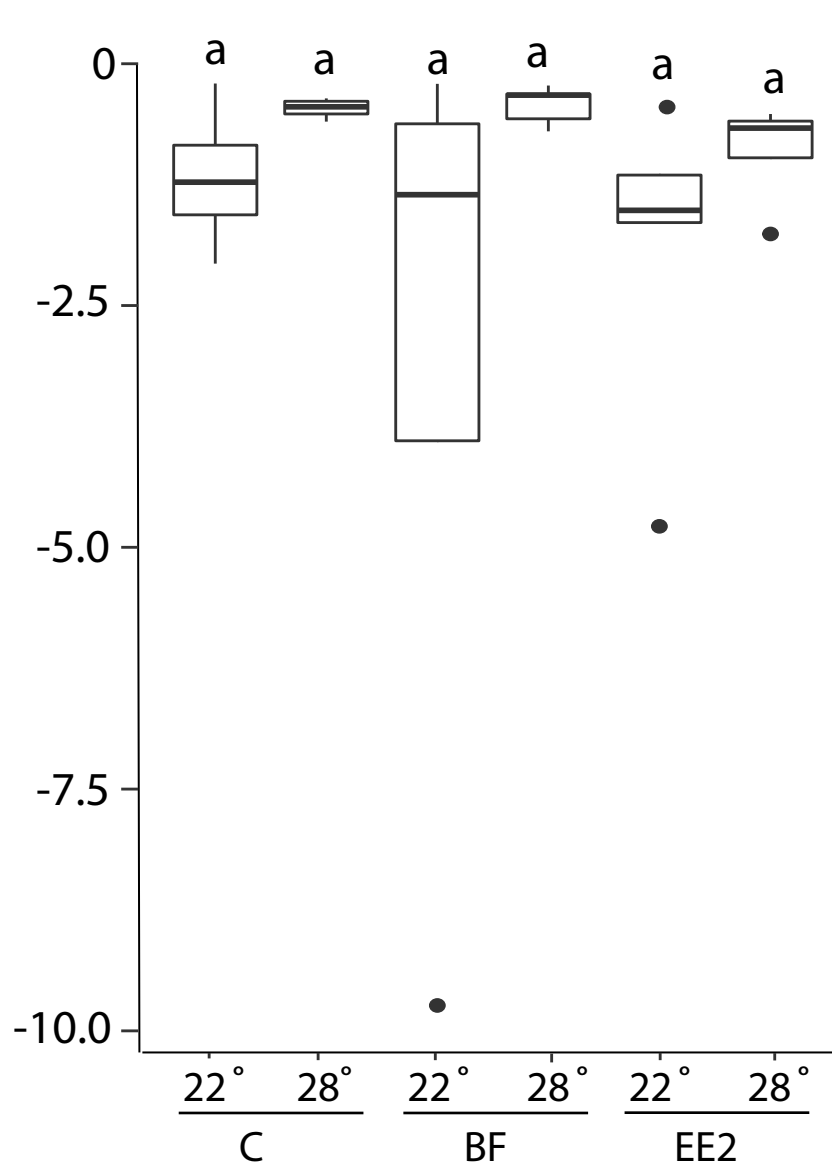

INHA

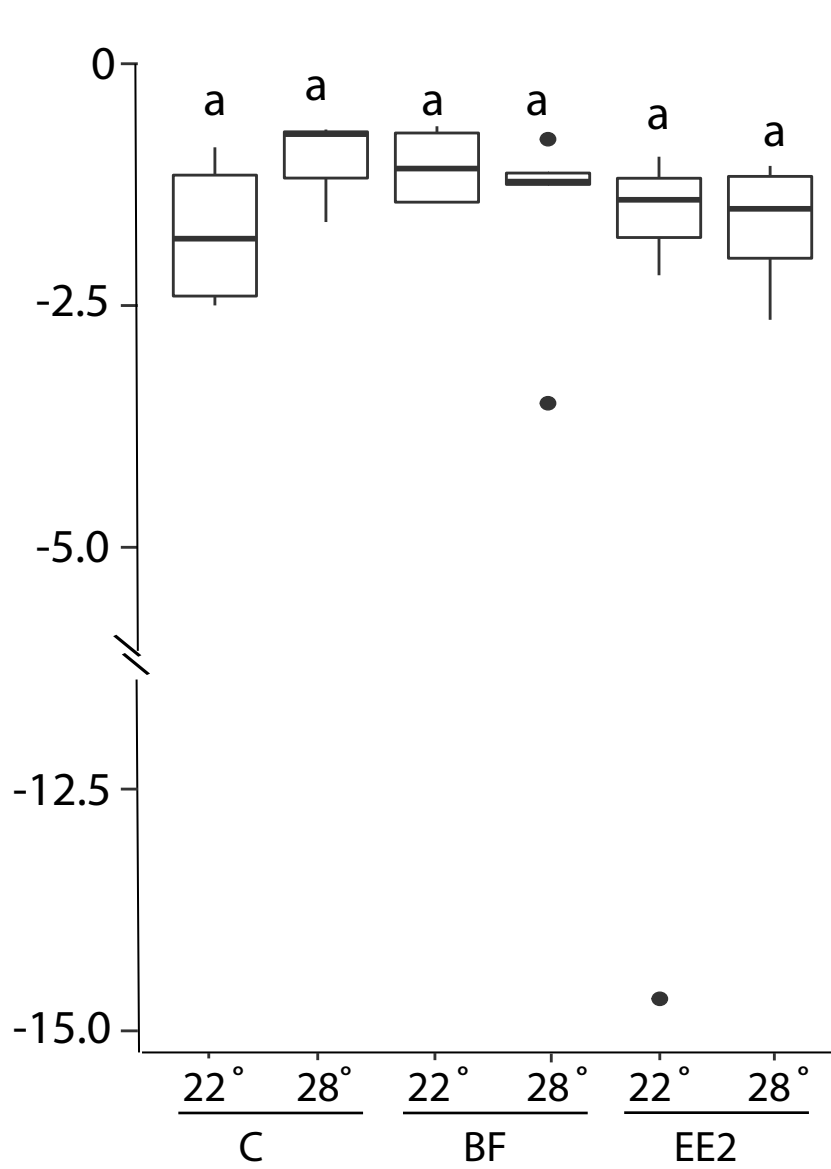

3β-HSD

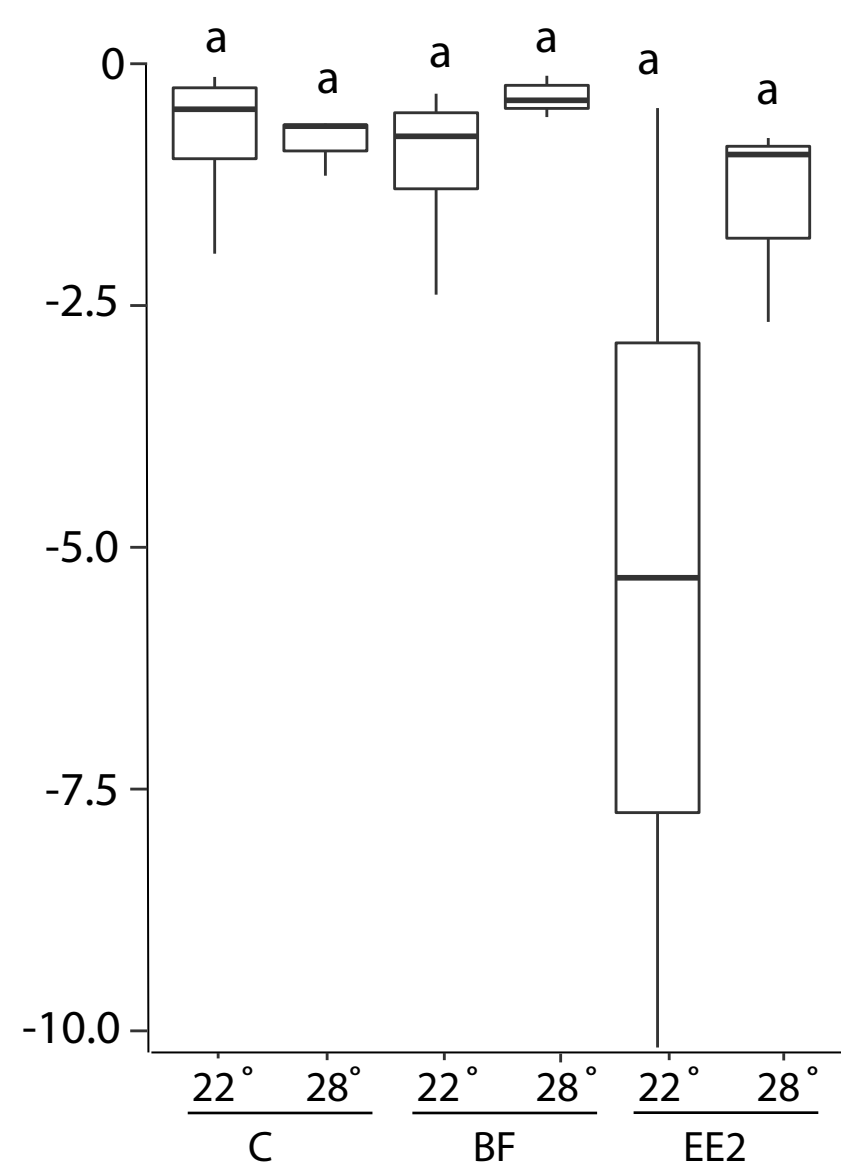

17β-HSD

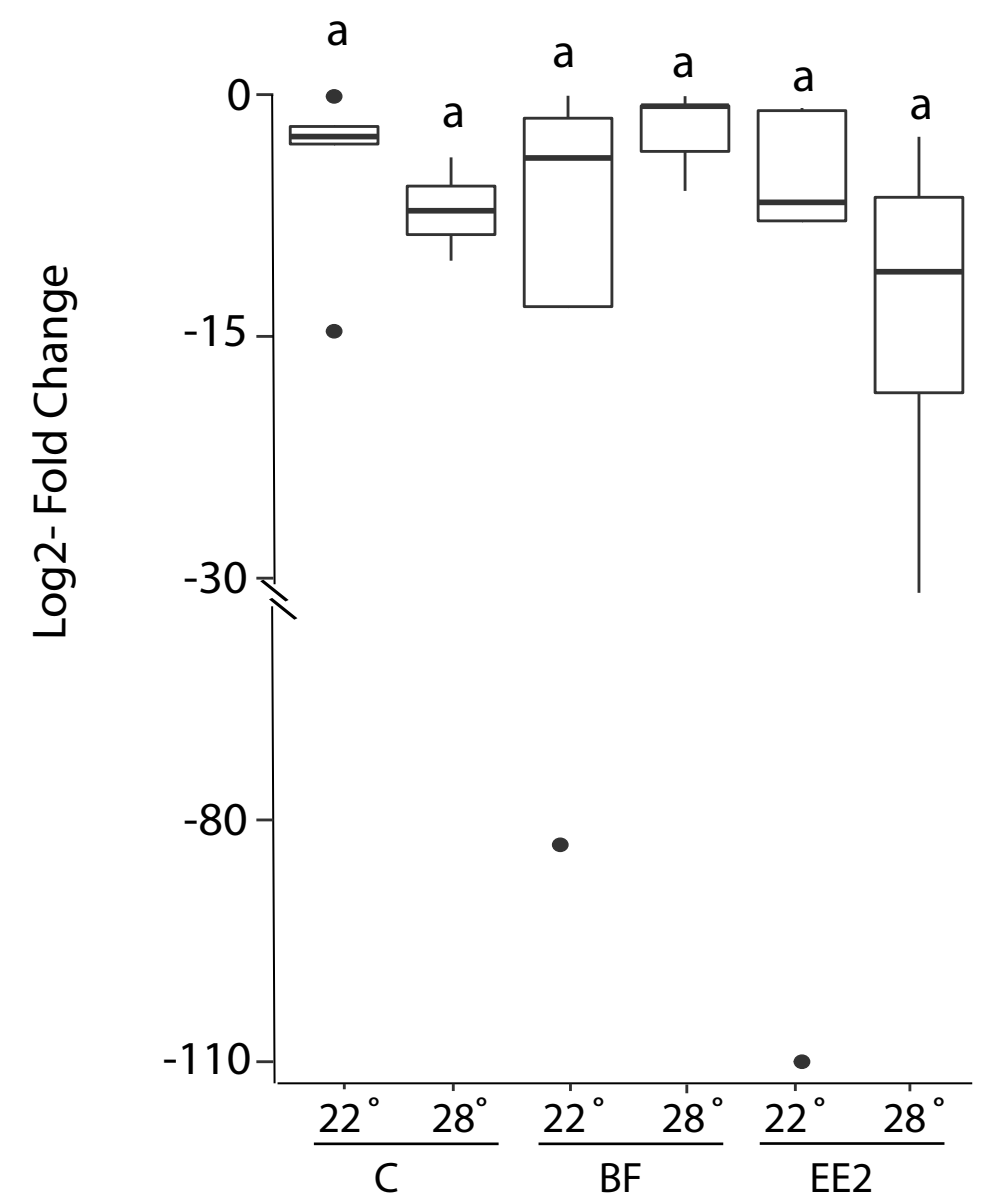

FSHR

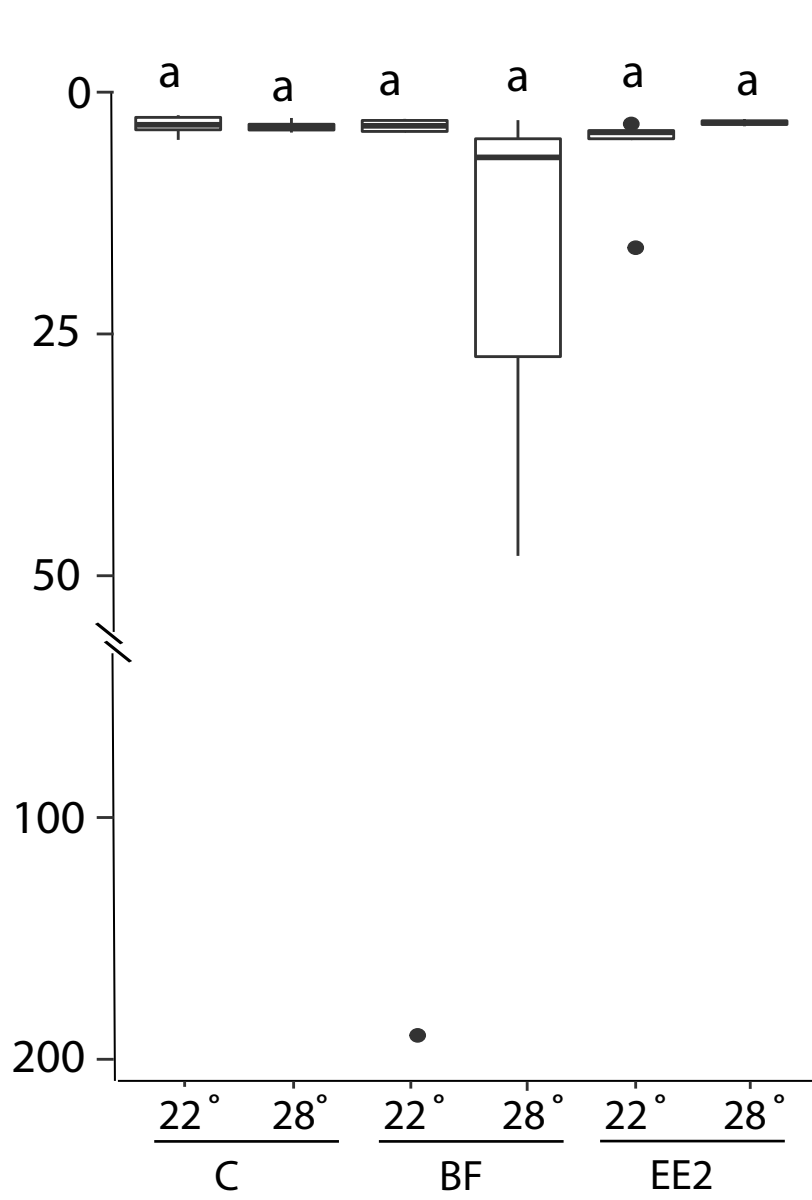

Arx

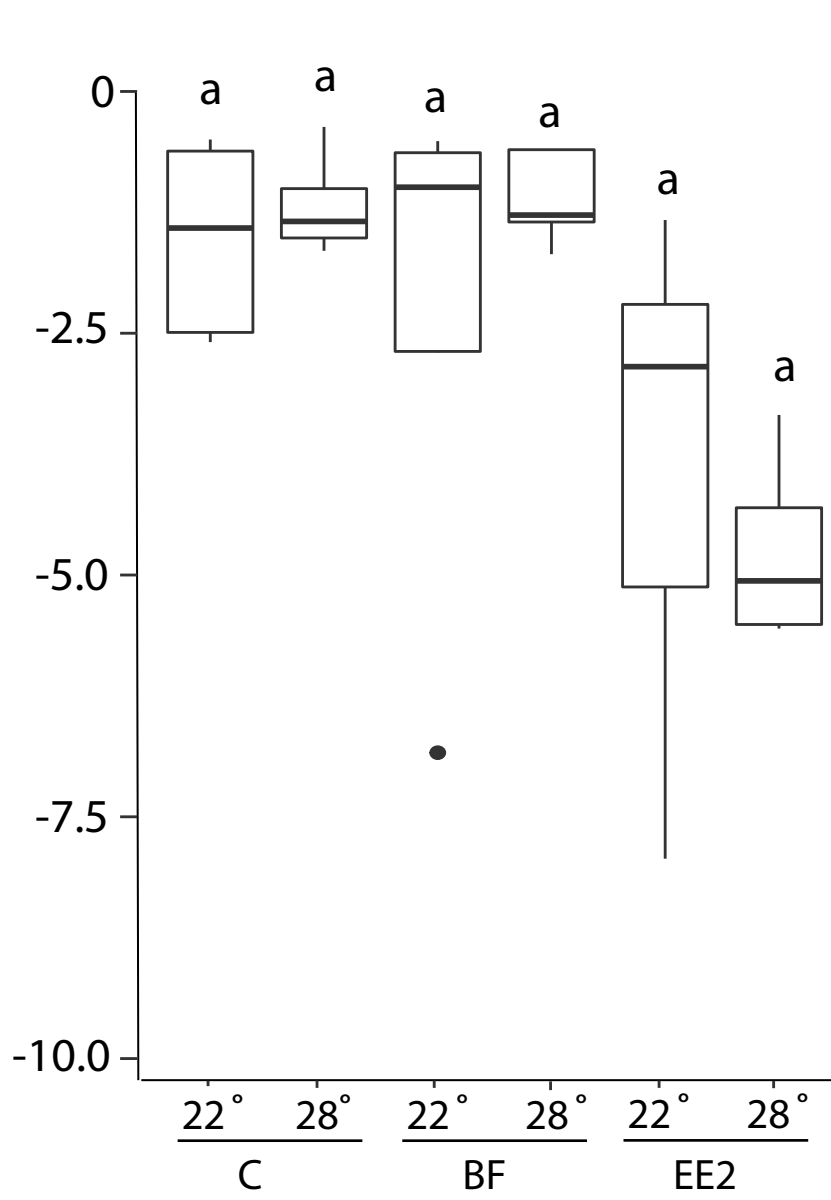

CYP19a

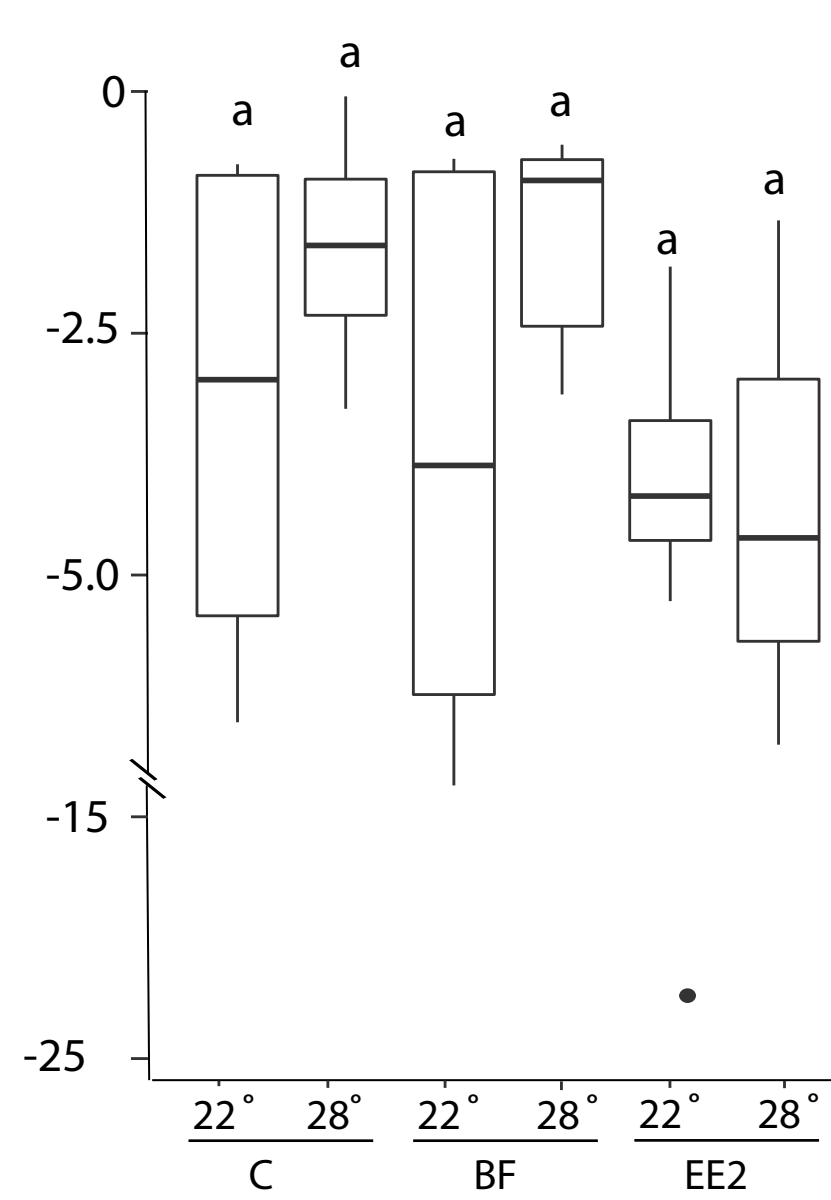

HSP90

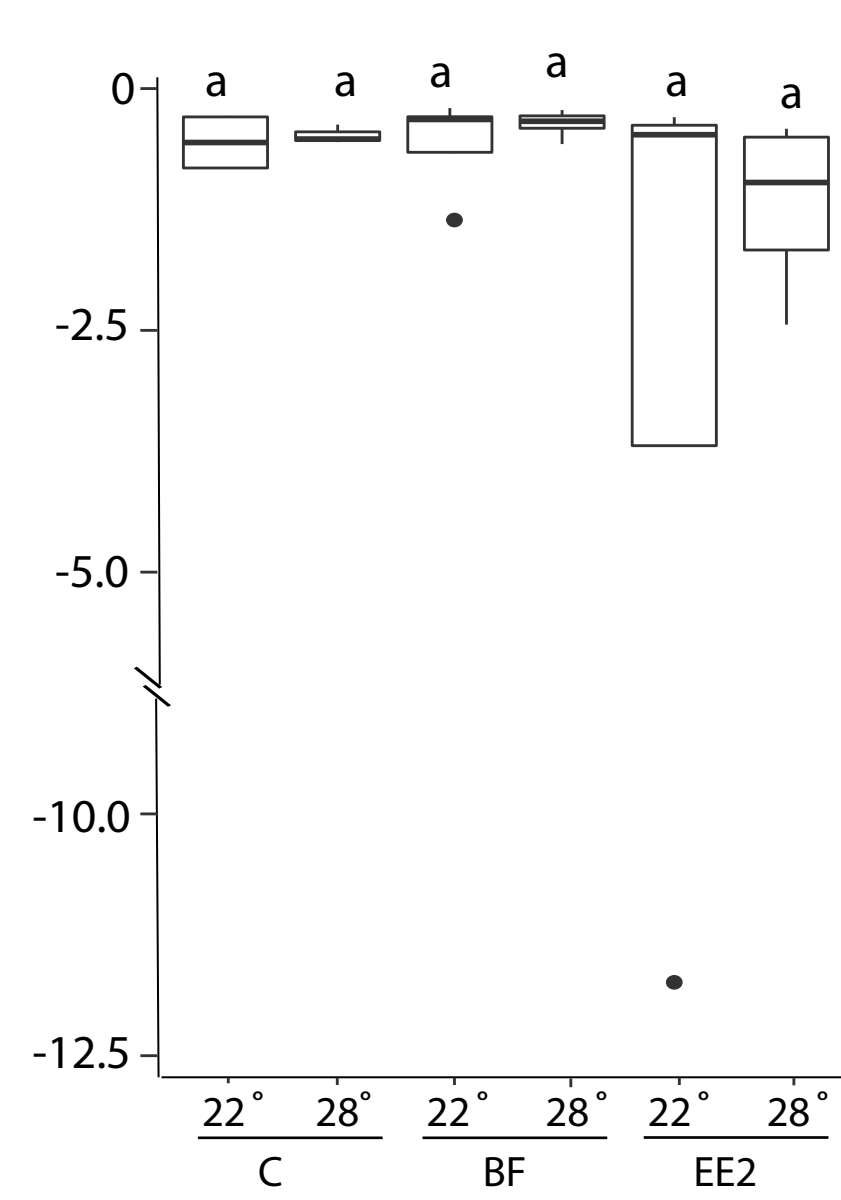

Supplement: Figure S3 [file peerj-07-6156-s005.pdf]

Relative gene expression of F1 testes

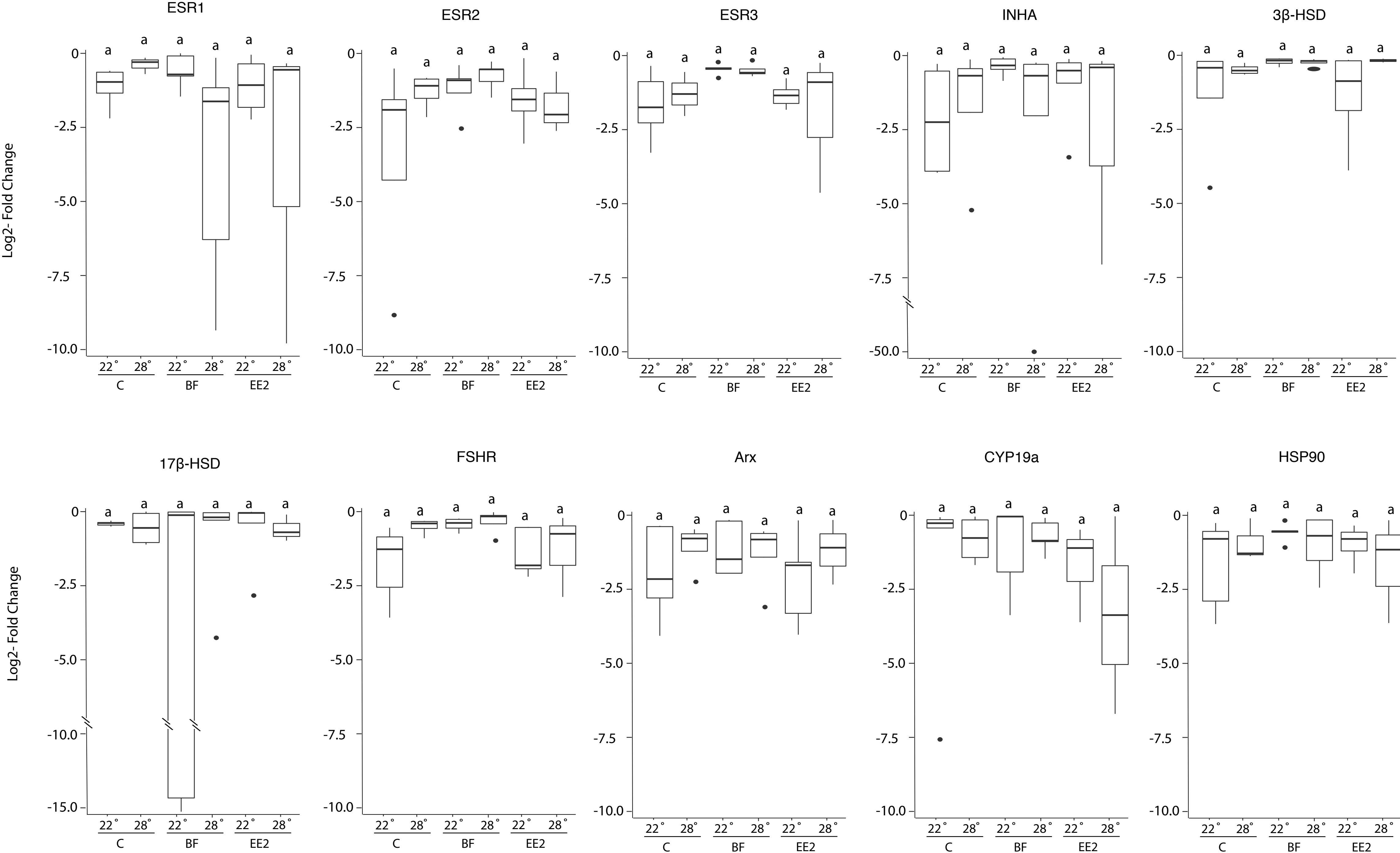

Supplement: Figure S4 [file peerj-07-6156-s006.pdf]
